# Supplementary material for: The role of solid solutions in iron phosphate-based electrodes for selective electrochemical lithium extraction
Source: Nat Commun. 2022 Aug 5;13:4579. doi: 10.1038/s41467-022-32369-y (PMC9355959; doi:10.1038/s41467-022-32369-y)
Supplement: Supplementary file 1 — Supporting information [file 41467_2022_32369_MOESM1_ESM.pdf]

Supplementary Information

The role of solid solutions in iron phosphate-based electrodes for selective electrochemical lithium extraction

Gangbin Yan[1], George Kim[2], Renliang Yuan[3], Eli Hoenig[1], Fengyuan Shi[4], Wenxiang Chen[3], Yu Han[1], Qian Chen[3], Jian-Min Zuo[3], Wei Chen[2] and Chong Liu[1,*]

[1]Pritzker School of Molecular Engineering, University of Chicago, Chicago, IL 60637, USA
[2]Department of Mechanical, Illinois Institute of Technology, Materials and Aerospace Engineering, Chicago, IL 60616, USA
[3]Department of Materials Science and Engineering, University of Illinois at Urbana-Champaign, Urbana, IL 61801, USA
[4]Electron Microscopy Core, Research Resources Center, University of Illinois Chicago, Chicago, IL 60612, USA
[*]Correspondence: chongliu@uchicago.edu

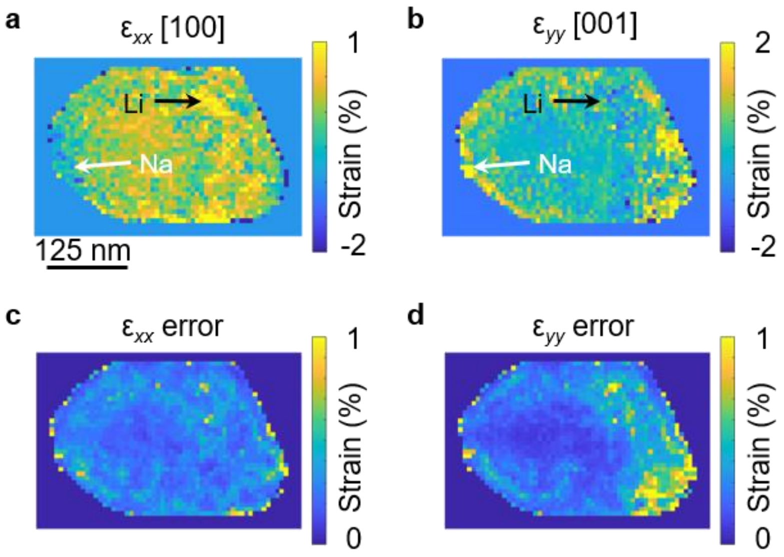

Supplementary Figure 1 Strain maps of the $LN(0.7)_{0.1C}$ particle along ε_{xx} [100] and ε_{yy} [001] directions converted from the lattice spacing maps with corresponding strain error. The measurement error is converted from uncertainty of diffraction peak detection. (Arrows are a guide-to-the-eye for the phase identification.)

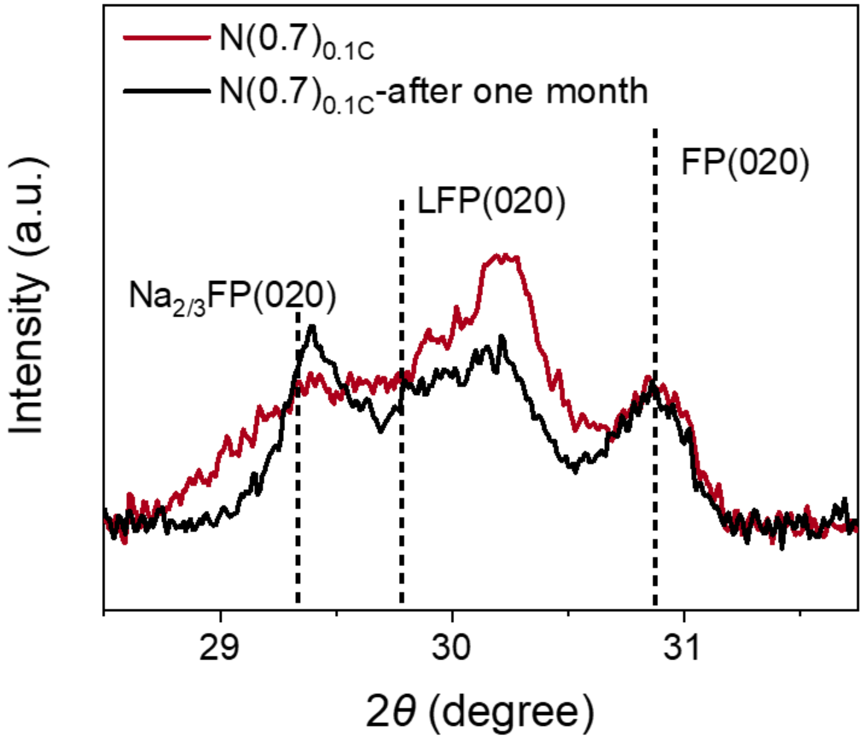

Supplementary Figure 2 XRD patterns of N(0.7)$_{0.1C}$ hosts right after Na-ion insertion and after storage in N_2/H_2O glovebox (< 1 ppm O_2) for one month.

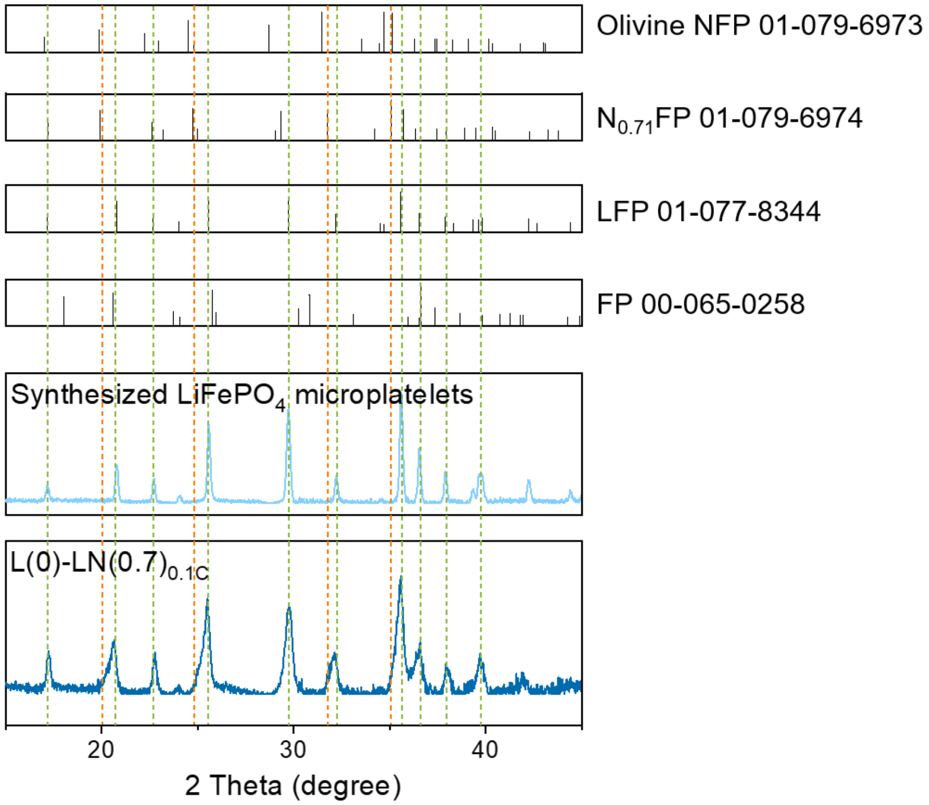

Supplementary Figure 3 XRD patterns of synthesized $LiFePO_4$ microplatelets and Li-Na co-intercalated $LN(0.7)_{0.1C}$ microplatelets. Green dashed lines denote the characteristic peaks of olivine $LiFePO_4$ phase (01-077-8344), and orange dashed lines denote the characteristic peaks of olivine $Na_{0.71}FePO_4$ intermediate phase (01-079-6974).

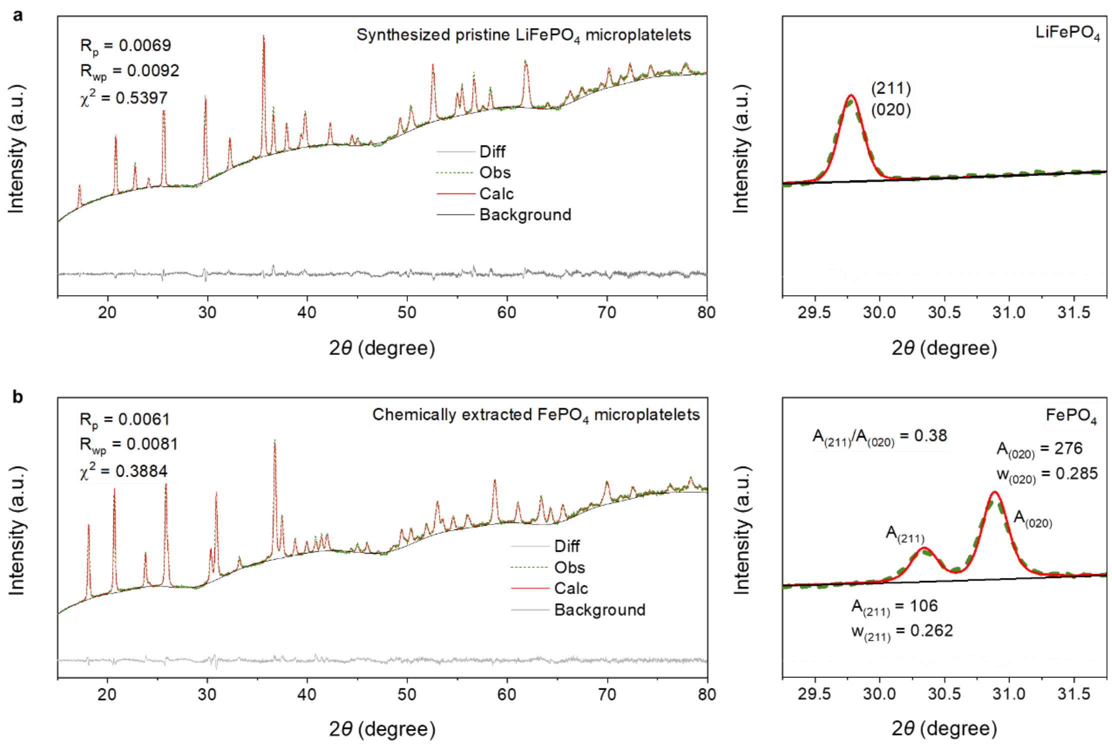

Supplementary Figure 4 Lebail refinement of (a) Synthesized $LiFePO_4$ and (b) Chemically Li-extracted $FePO_4$.

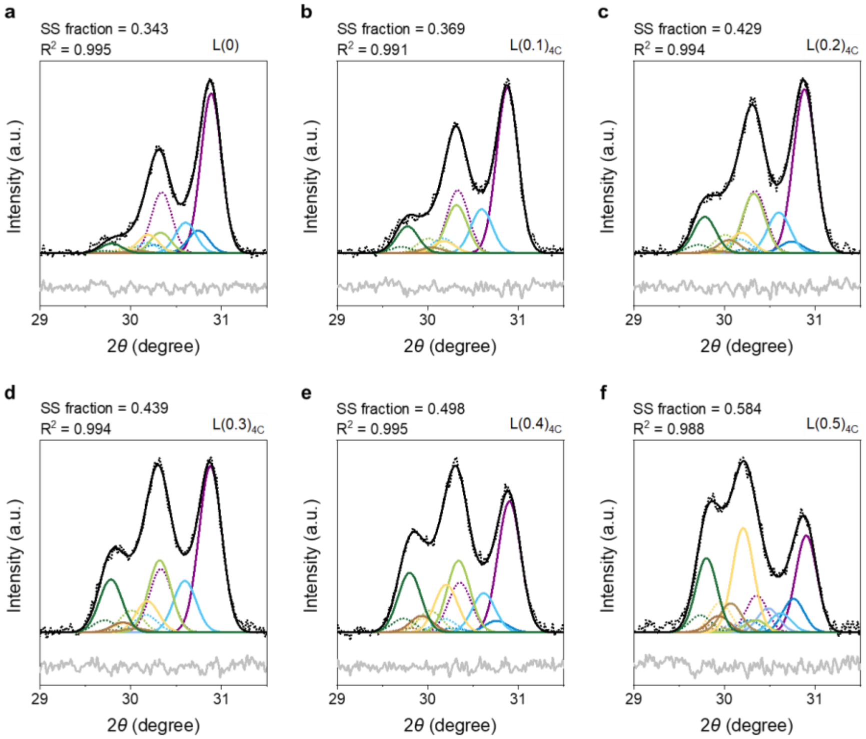

Supplementary Figure 5 Deconvoluted XRD patterns of (a) Chemically Li-extracted $FePO_4$ before seeding L(0), (b) $L(0.1)_{4C}$, (c) $L(0.2)_{4C}$, (d) $L(0.3)_{4C}$, (e) $L(0.4)_{4C}$ and (f) $L(0.5)_{4C}$ for Synthesized-$FePO_4$ particles on carbon cloth substrate from one representative sample.

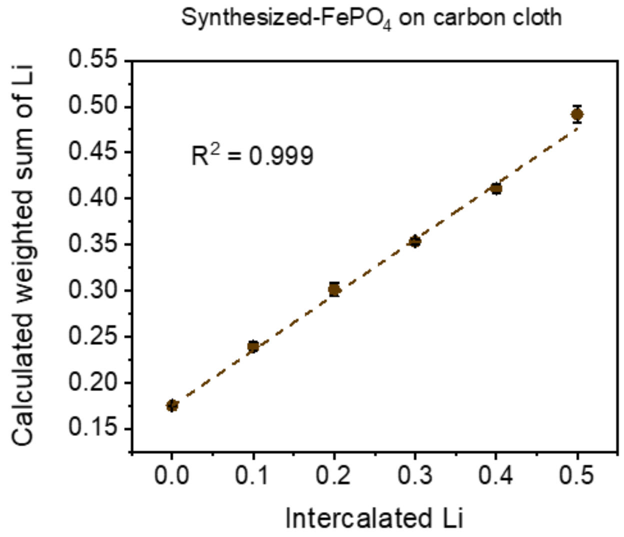

Supplementary Figure 6 Calculated weighted sum of Li from XRD fittings versus the electrochemically intercalated Li amount with the use of Synthesized-$FePO_4$ particles on carbon cloth. (Error bars representing the standard deviation of three replicate measurements.)

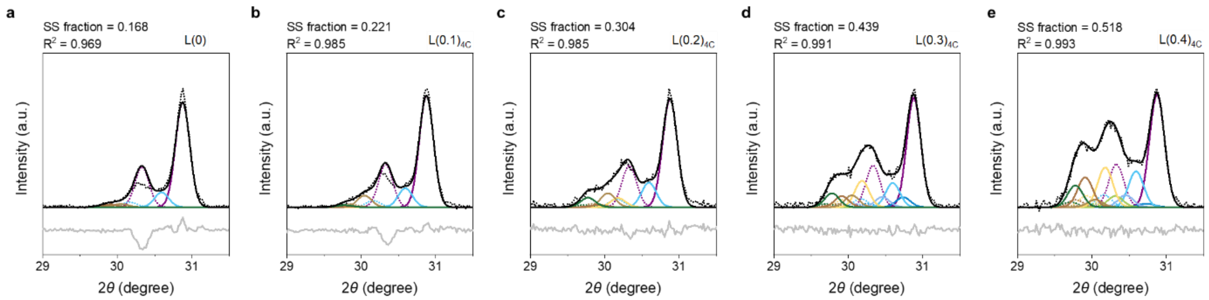

Supplementary Figure 7 Deconvoluted XRD patterns of (a) Chemically Li-extracted $FePO_4$ before seeding L(0), (b) L(0.1)$_{4C}$, (c) L(0.2)$_{4C}$ (d) L(0.3)$_{4C}$, and (e) L(0.4)$_{4C}$ for Synthesized-$FePO_4$ particles on glassy carbon substrate from one representative sample.

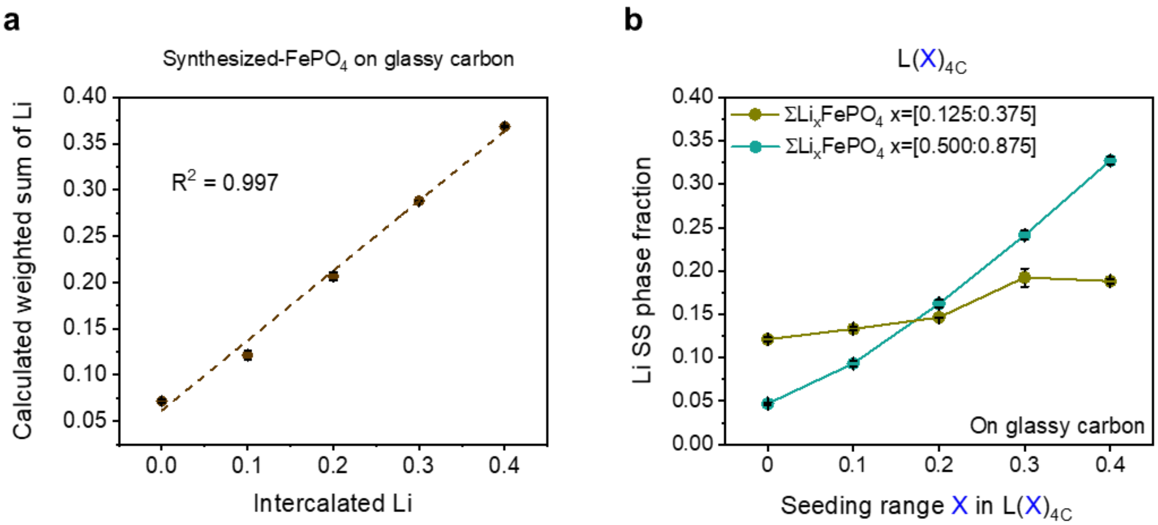

Supplementary Figure 8 (a) Calculated weighted sum of Li from XRD fittings versus the electrochemically intercalated Li ions amount with the use of Synthesized-$FePO_4$ particles on glassy carbon; (b) High-Li SS fractions (Li_xFePO_4, x = 0.500/0.625/0.750/0.875) and low-Li SS fractions (Li_xFePO_4, x = 0.125/0.250/0.375) under the same seeding rate 4C (588 mA/g) with different seeding ranges $L(0/0.1/0.2/0.3/0.4)_{4C}$ collected on glassy carbon. (Error bars representing the standard deviation of three replicate measurements.)

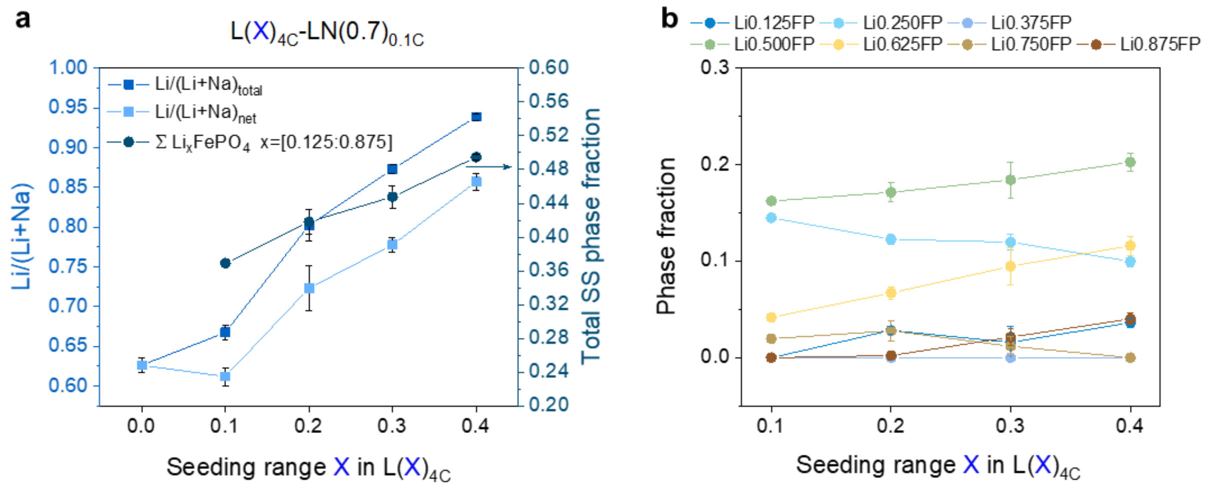

Supplementary Figure 9 (a) Total-SS fractions under the same seeding rate 4C (588 mA/g) with different seeding ranges $L(0/0.1/0.2/0.3/0.4)_{4C}$ and measured $Li/(Li+Na)_{total}$ and $Li/(Li+Na)_{net}$ ratios after 0.1C (14.7 mA/g) co-intercalation $L(X)_{4C}$-$LN(0.7)_{0.1C}$. (b) Individual phase fractions under the same seeding rate 4C (588 mA/g) with different seeding ranges $L(0/0.1/0.2/0.3/0.4)_{4C}$.

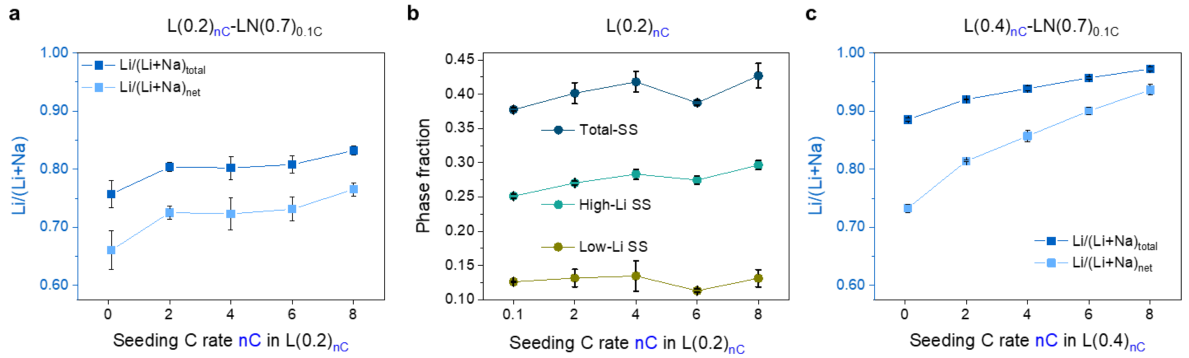

Supplementary Figure 10 (a) Recovered $Li/(Li+Na)_{total}$ and $Li/(Li+Na)_{net}$ of $L(0.2)_{0.1/2/4/6/8C}$-$LN(0.7)_{0.1C}$. (b) SS fractions under different seeding C rates with the same 20% seeding range $L(0.2)_{0.1/2/4/6/8C}$. (c) Recovered $Li/(Li+Na)_{total}$ and $Li/(Li+Na)_{net}$ of $L(0.4)_{0.1/2/4/6/8C}$-$LN(0.7)_{0.1C}$.

(Noting that 1C is equivalent to 147 mA/g)

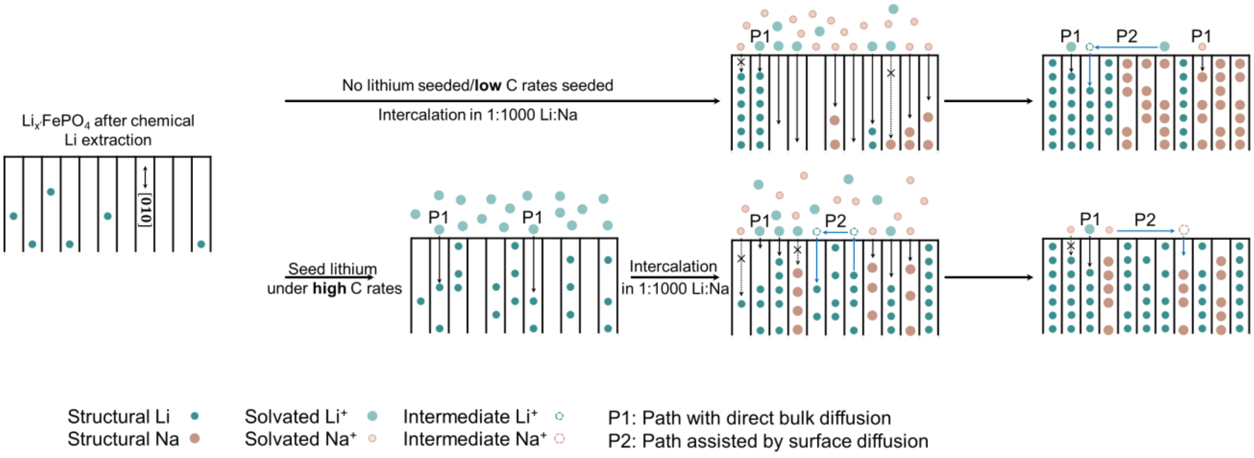

Supplementary Figure 11 Schematic illustration of Li-Na co-intercalation pathways for chemically Li extracted $Li_{x'}FePO_4$ hosts (x' denotes the remnant quantity of Li in the structure due to the defect), low-rate Li pre-seeded hosts, and high-rate Li pre-seeded hosts.

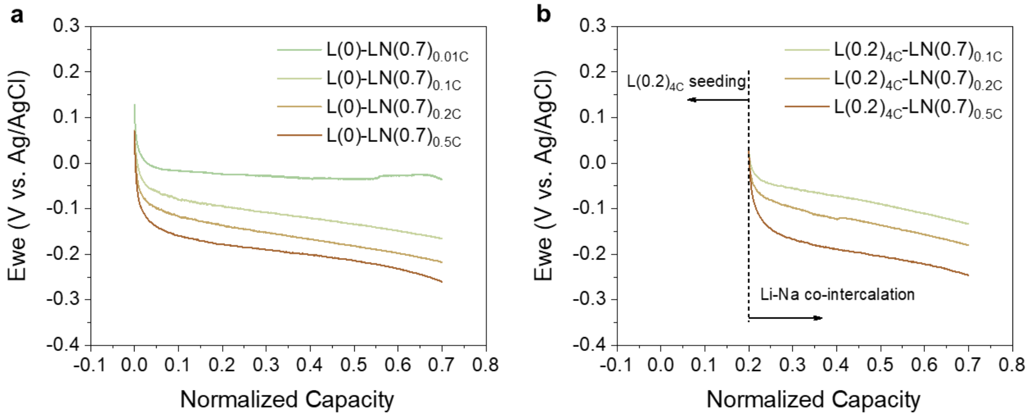

Supplementary Figure 12 Intercalation curves of (a) L(0)-LN(0.7)$_{0.01/0.1/0.2/0.5C}$ and (b) L(0.2)$_{4C}$-LN(0.7)$_{0.1/0.2/0.5C}$. Without (L(0)) or with 4C (588 mA/g) Li seeding (L(0.2)$_{4C}$), all the working electrodes, paired with $NaFePO_4$ counter electrodes and Ag|AgCl|KCl (4.0 M) reference electrodes, would undergo intercalation in a three-neck round-bottomed flask containing 500 mL synthetic brine solutions (1 mM LiCl and 1 M NaCl mixed solution) at room temperature (20 ~ 25 °C). Different intercalation C rates (0.01C, 0.1C, 0.2C, or 0.5C; 0.1C equals to 14.7 mA/g) were used until 70% of the total capacity. N_2 (purity > 99.998%) was continuously bubbled into the solution to avoid side reactions caused from dissolved O_2.

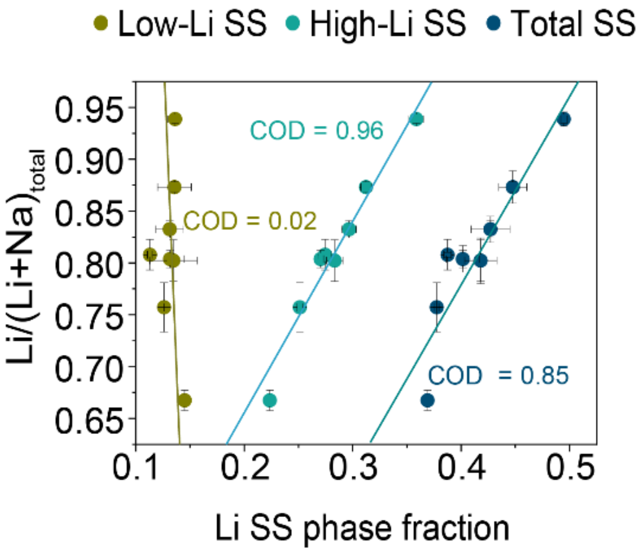

Supplementary Figure 13 Li/(Li+Na)$_{total}$ vs. low-Li/high-Li/total-Li SS fractions and corresponding coefficients of determination (COD). (Error bars representing the standard deviation of three replicate measurements)

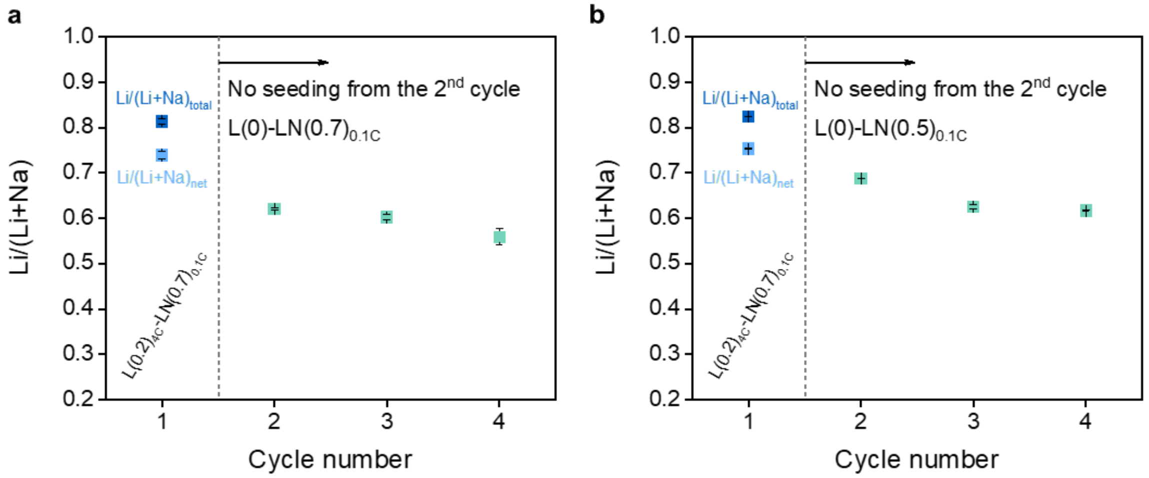

Supplementary Figure 14 Li/(Li+Na)$_{net}$ for the multi-intercalation stability test using 20% Li seeding for the 1st cycle and skipping Li seeding for the following cycles. (a) 70% capacity usage from the 2nd cycle, L(0)-LN(0.7)$_{0.1C}$. (b) 50% capacity usage from the 2nd cycle, L(0)-LN(0.5)$_{0.1C}$.

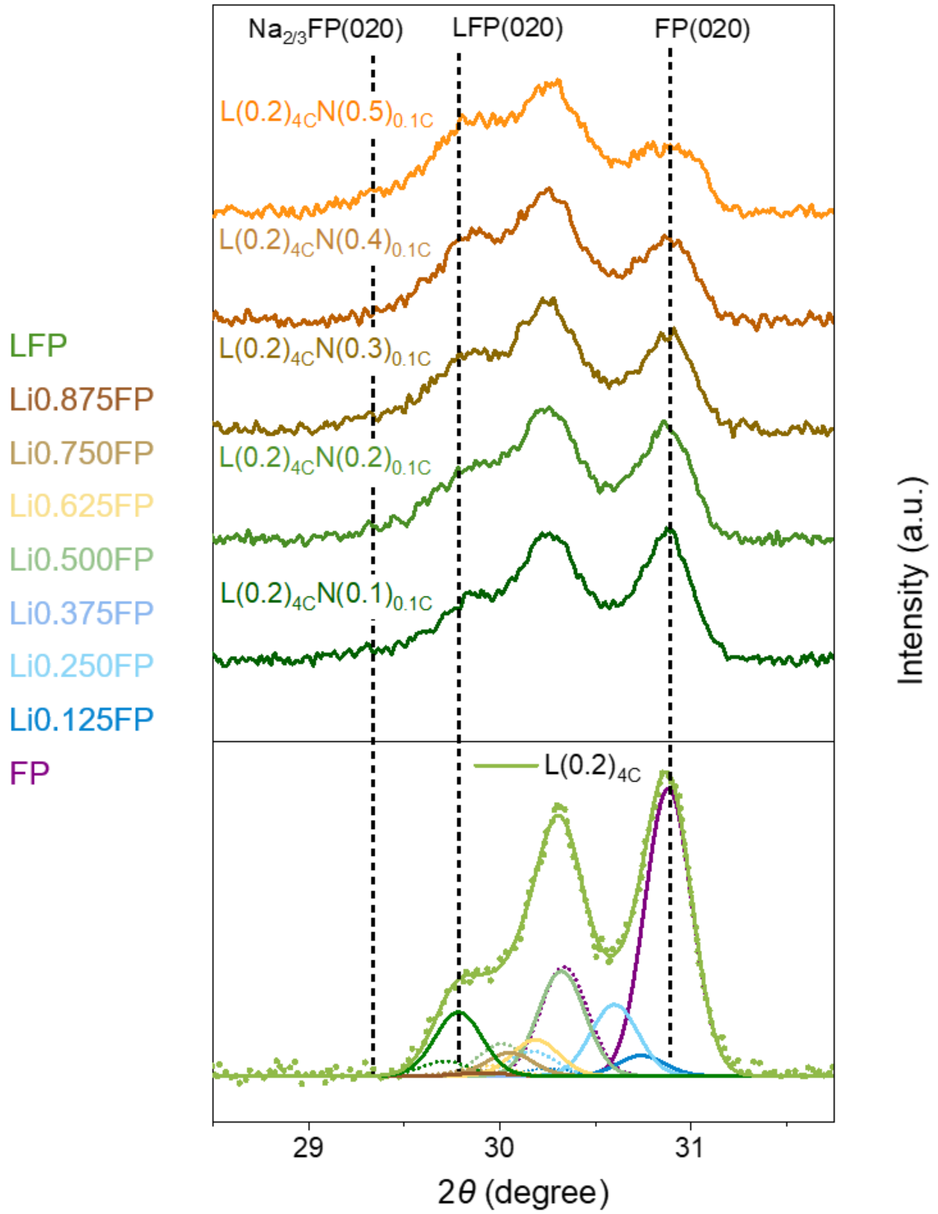

Supplementary Figure 15 Ex situ XRD evolution of $L(0.2)_{4C}$ preseed host during Na insertion $L(0.2)_{4C}N(0/0.1/0.2/0.3/0.4/0.5)_{0.1C}$. Color texts denote the deconvoluted Li SS phases for $L(0.2)_{4C}$. (Dotted lines: raw data; Solid lines: fit)

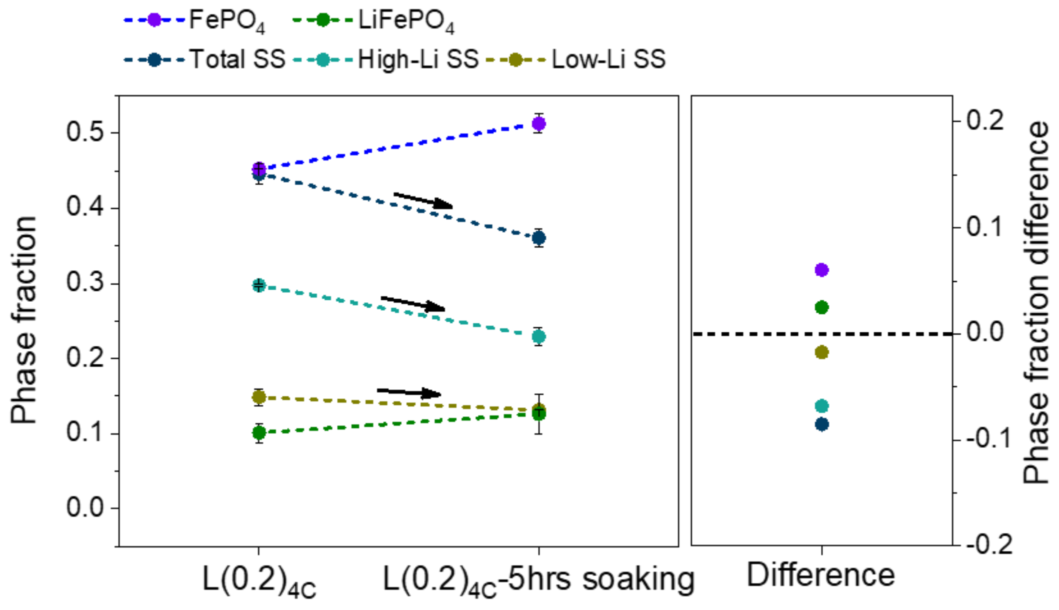

Supplementary Figure 16 Fitted phase fractions for L(0.2)$_{4C}$ microplatelets before and after 5hrs soaking in 1M NaCl$_{(aq)}$. (Error bars representing the standard deviation of three replicate measurements.)

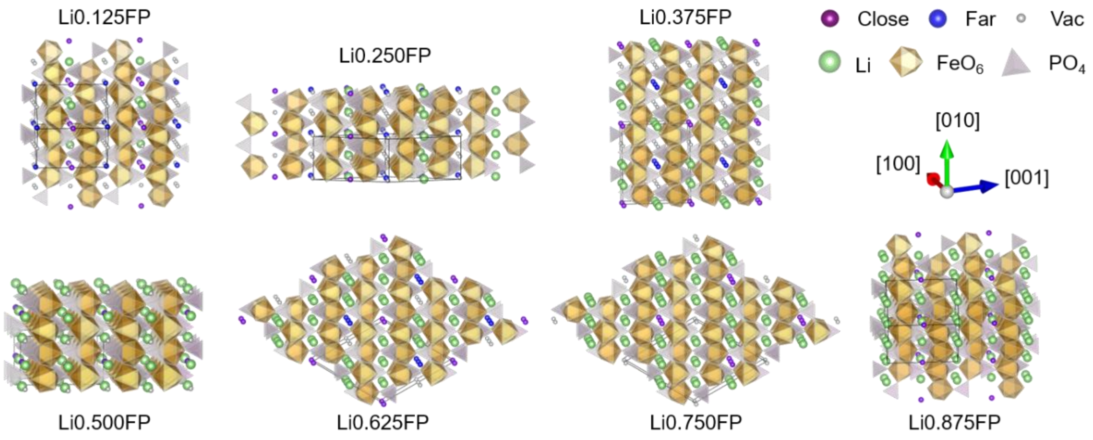

Supplementary Figure 17 Structures for the seven intermediate phases with either close or far intercalation positions labeled.

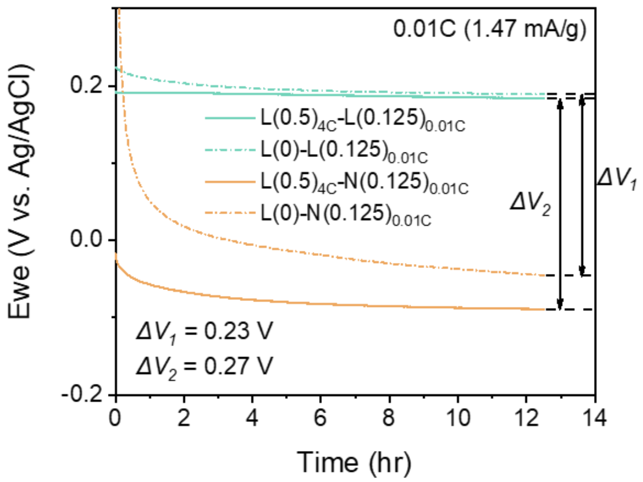

Supplementary Figure 18 Intercalation curves of chemically Li-extracted hosts (L(0); dash-dot lines) and 4C (588 mA/g) 50% Li pre-seeded hosts ($L(0.5)_{4C}$; solid lines) in 60 mL either 1 M LiCl (green) or 1 M NaCl (orange) aqueous solution until 12.5% of capacity are used under 0.01C (1.47 mA/g) at room temperature (20 ~ 25 °C). The reference electrode is always Ag|AgCl|KCl (4.0 M). To avoid co-intercalation, in 1 M LiCl aqueous solution, the counter electrode is $LiFePO_4$; while in 1 M NaCl aqueous solution, the counter electrode is $NaFePO_4$. N_2 (purity > 99.998%) was continuously bubbled into the solution to avoid side reactions caused from dissolved O_2.

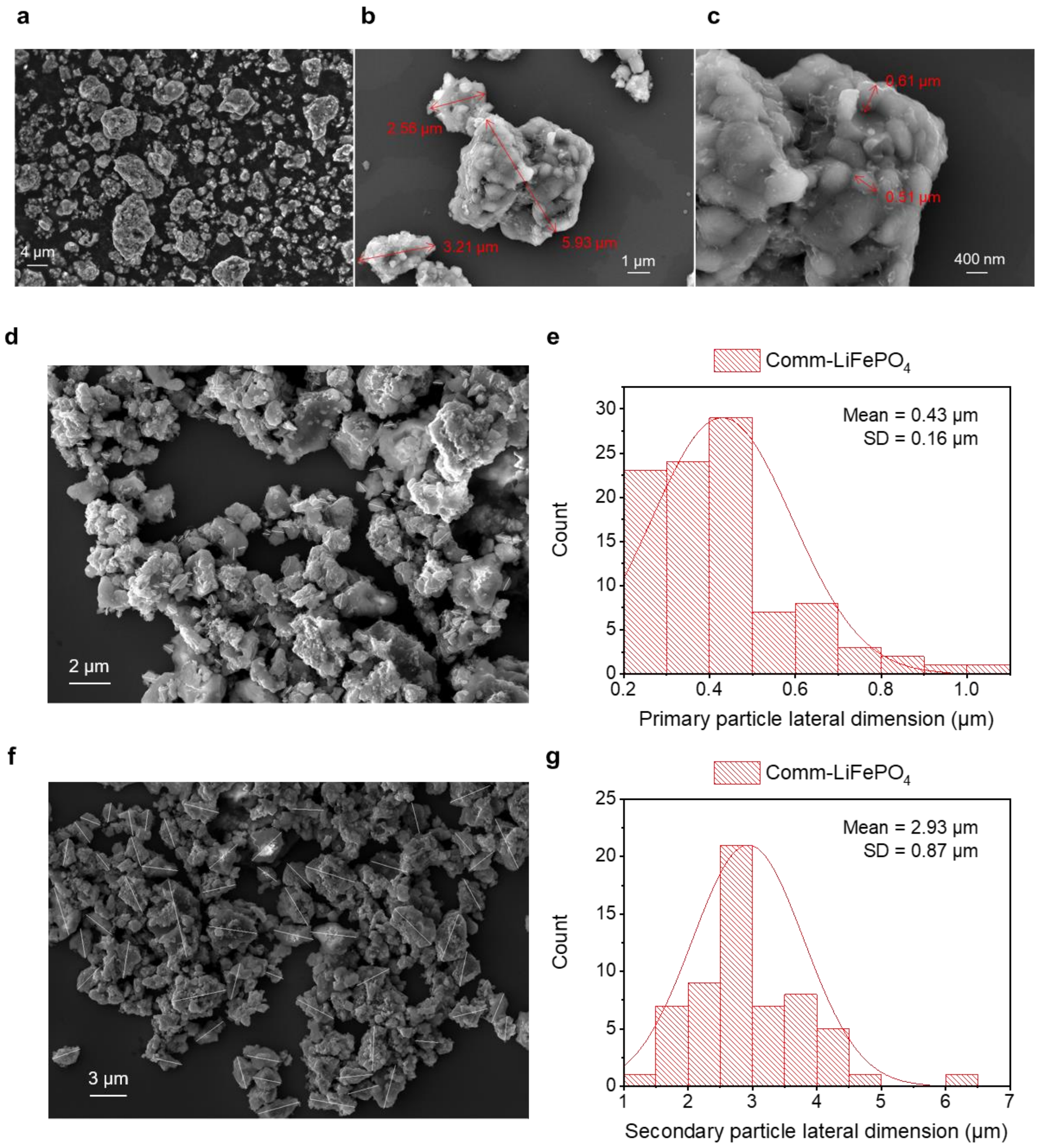

Supplementary Figure 19 SEM images for Comm-$LiFePO_4$ particles. (a) Comm-$LiFePO_4$ particles dropped on carbon tape, (b) Magnified SEM for Comm-$LiFePO_4$ secondary particles dispersed on Si wafer, (c) Magnified SEM for Comm-$LiFePO_4$ primary particles on Si, additional SEM images and corresponding lateral distribution summary of primary (d, e) and secondary (f, g) Comm-$LiFePO_4$ particles dispersed on Si wafer. (Only the isolated and fully exposed particles were counted; the thin white lines in the SEM images denoted the measured length.)

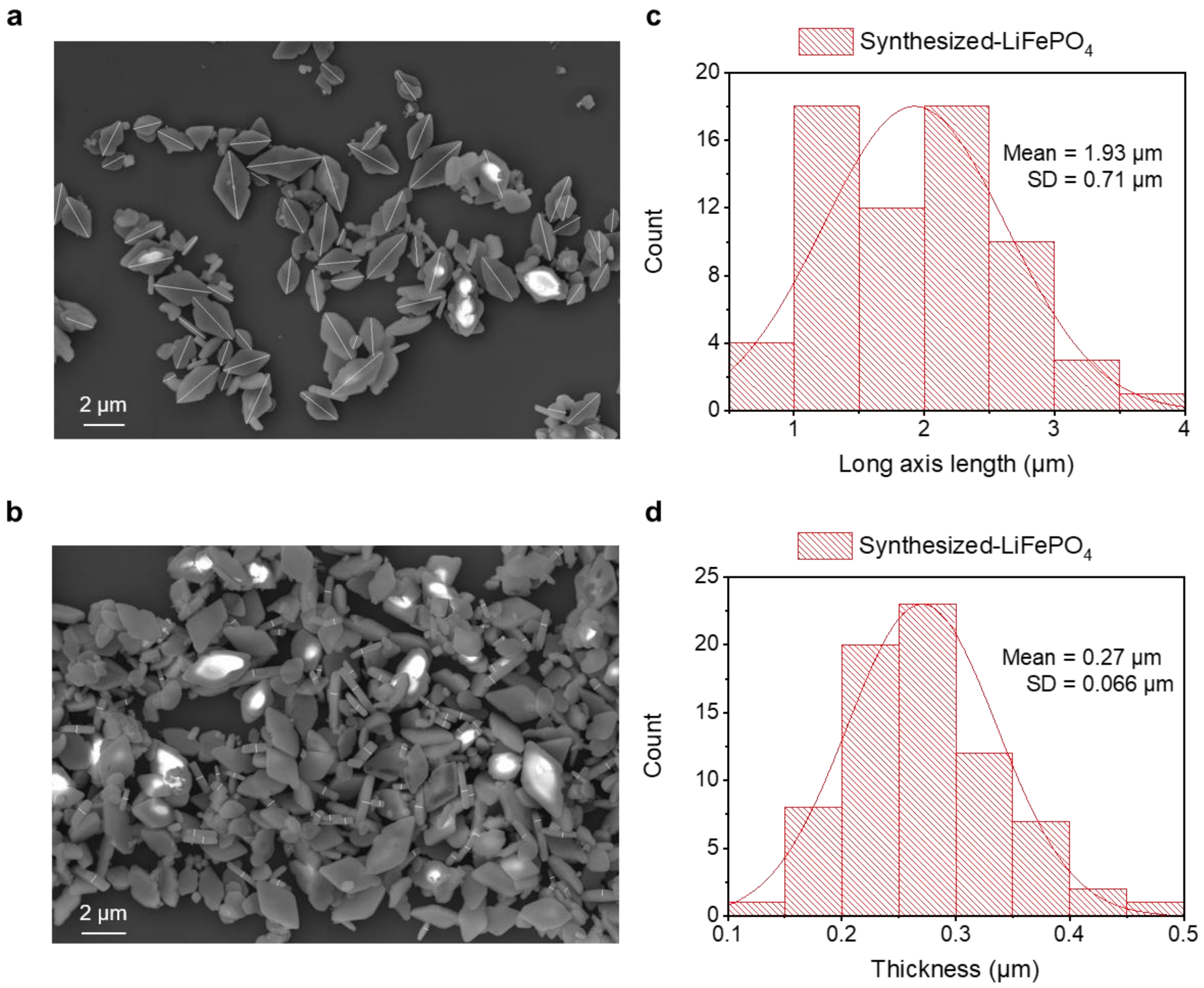

Supplementary Figure 20 SEM images of Synthesized-$LiFePO_4$ particles (a, b) with corresponding particle dimension distribution along the long axis (c) or thickness (d). (Only the isolated and fully exposed particles were counted; the thin white lines in the SEM images denoted the measured length.)

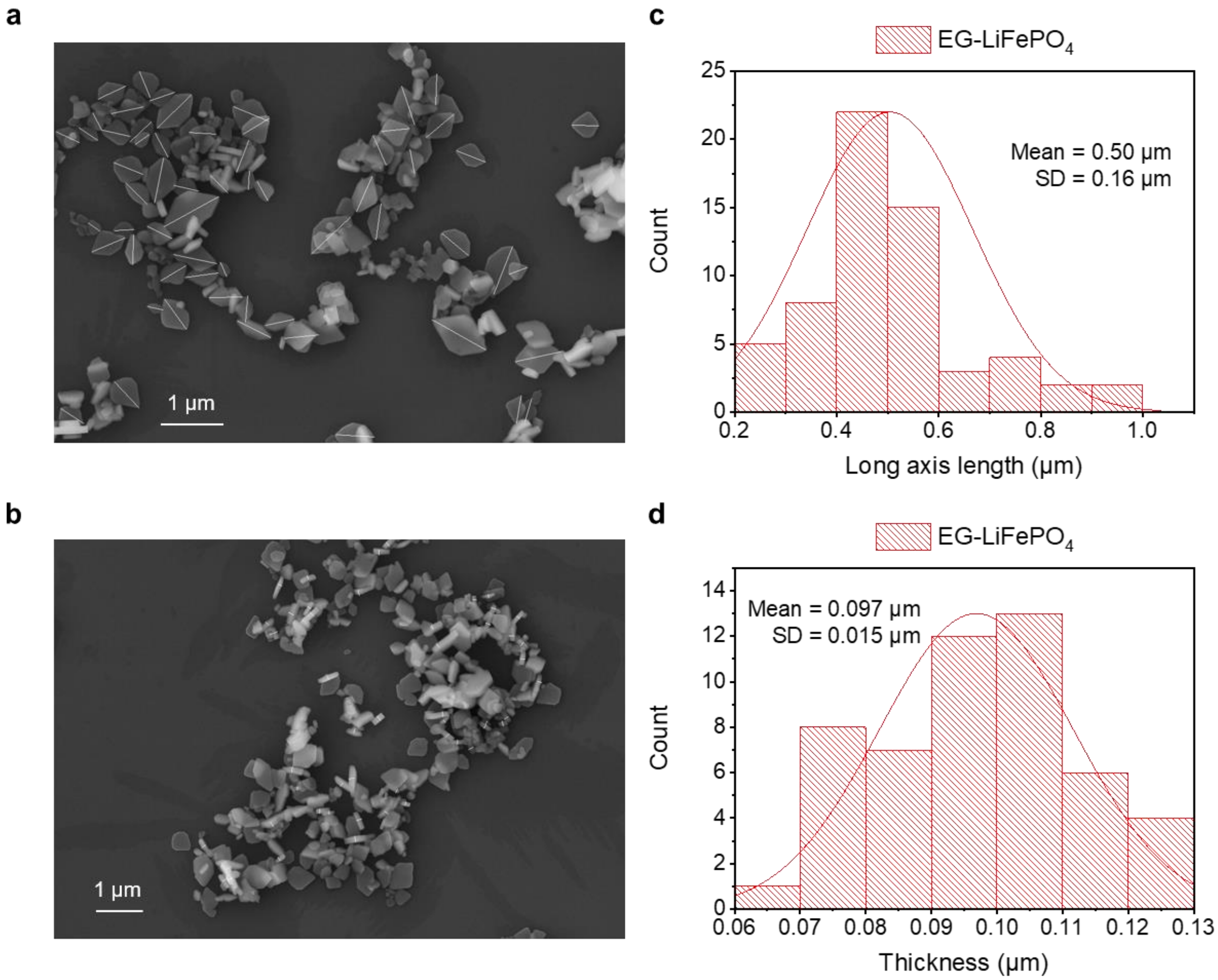

Supplementary Figure 21 SEM images of EG-$LiFePO_4$ particles (a, b) with corresponding particle dimension distribution along the long axis (c) or thickness (d). (Only the isolated and fully exposed particles were counted; the thin white lines in the SEM images denoted the measured length.)

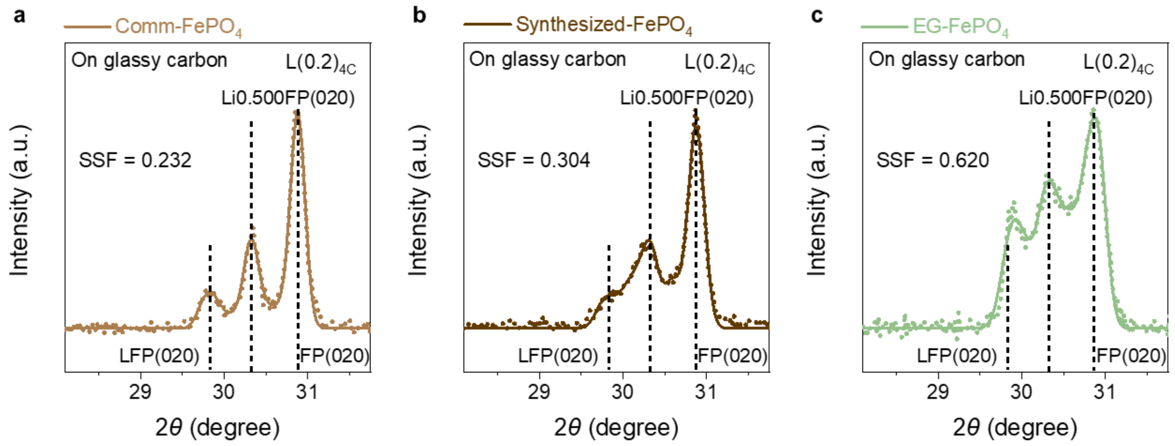

Supplementary Figure 22 Example XRD patterns of (a) Comm-$FePO_4$, (b) Synthesized-$FePO_4$, and **(c)** EG-$FePO_4$ electrodes after 20% of Li seeding under 4C collected on glassy carbon. For Comm-$FePO_4$, 135 mA/g equals a rate of 1C; for Synthesized-$FePO_4$, 147 mA/g equals a rate of 1C; for EG-$FePO_4$, 125 mA/g equals a rate of 1C (Dotted lines: raw data; Solid lines: fit).

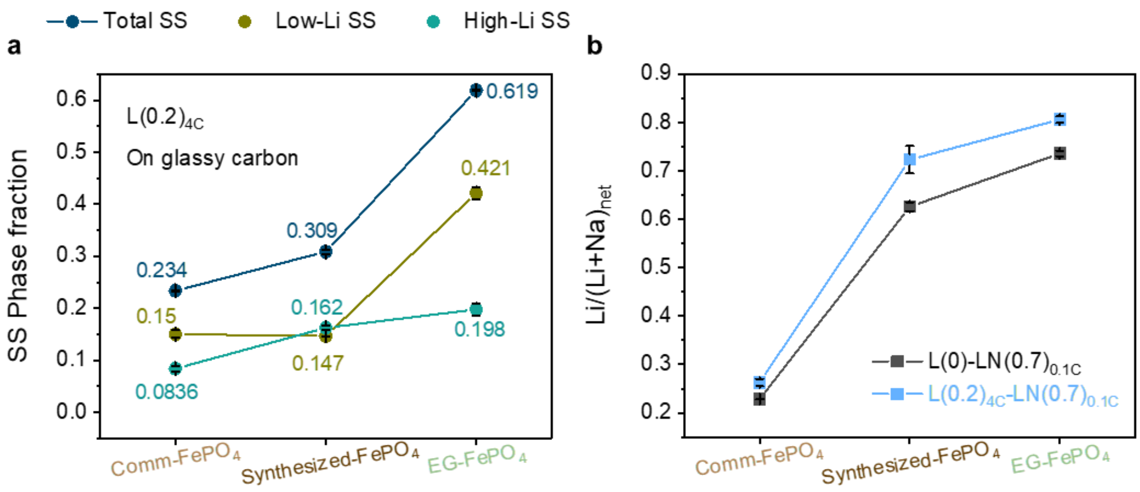

Supplementary Figure 23 (a) Total SS, Low-Li SS, and High-Li SS fractions for the three different $FePO_4$ samples under the same seeding rate 4C (540 mA/g for Comm-$FePO_4$; 588 mA/g for Synthesized-$FePO_4$; 500 mA/g for EG-$FePO_4$) with the 20% seeding range $L(0.2)_{4C}$. **(b)** Recovered $Li/(Li+Na)_{net}$ of the three electrodes until 70% of the total capacity used under 0.1C, with either initial 20% of Li seeding under 4C ($L(0.2)_{4C}$-$LN(0.7)_{0.1C}$) or without any initial seeding process ($L(0)$-$LN(0.7)_{0.1C}$). (Error bars represent the standard deviation of three replicate measurements.)

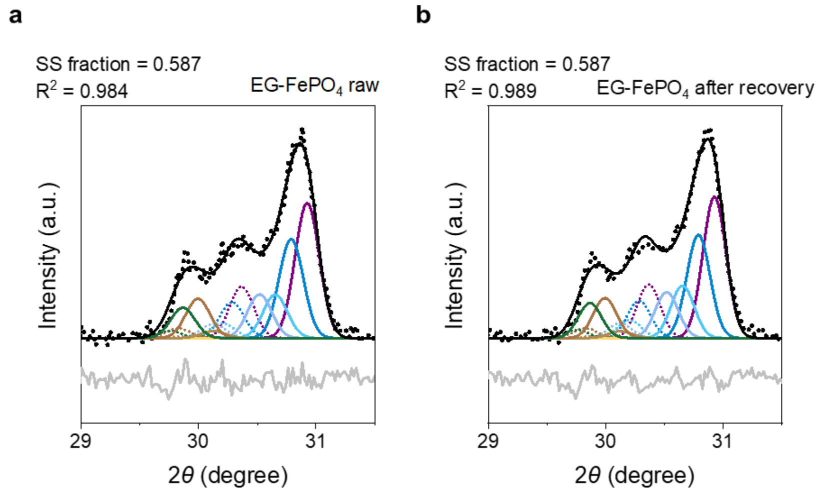

Supplementary Figure 24 Deconvoluted XRD patterns of EG-$FePO_4$ on glassy carbon (a) raw electrodes, (b) after Li recovery, and (c) After 4C 20% Li seeding, $L(0.2)_{4C}$. (Dotted lines: raw data; Solid lines: fit).

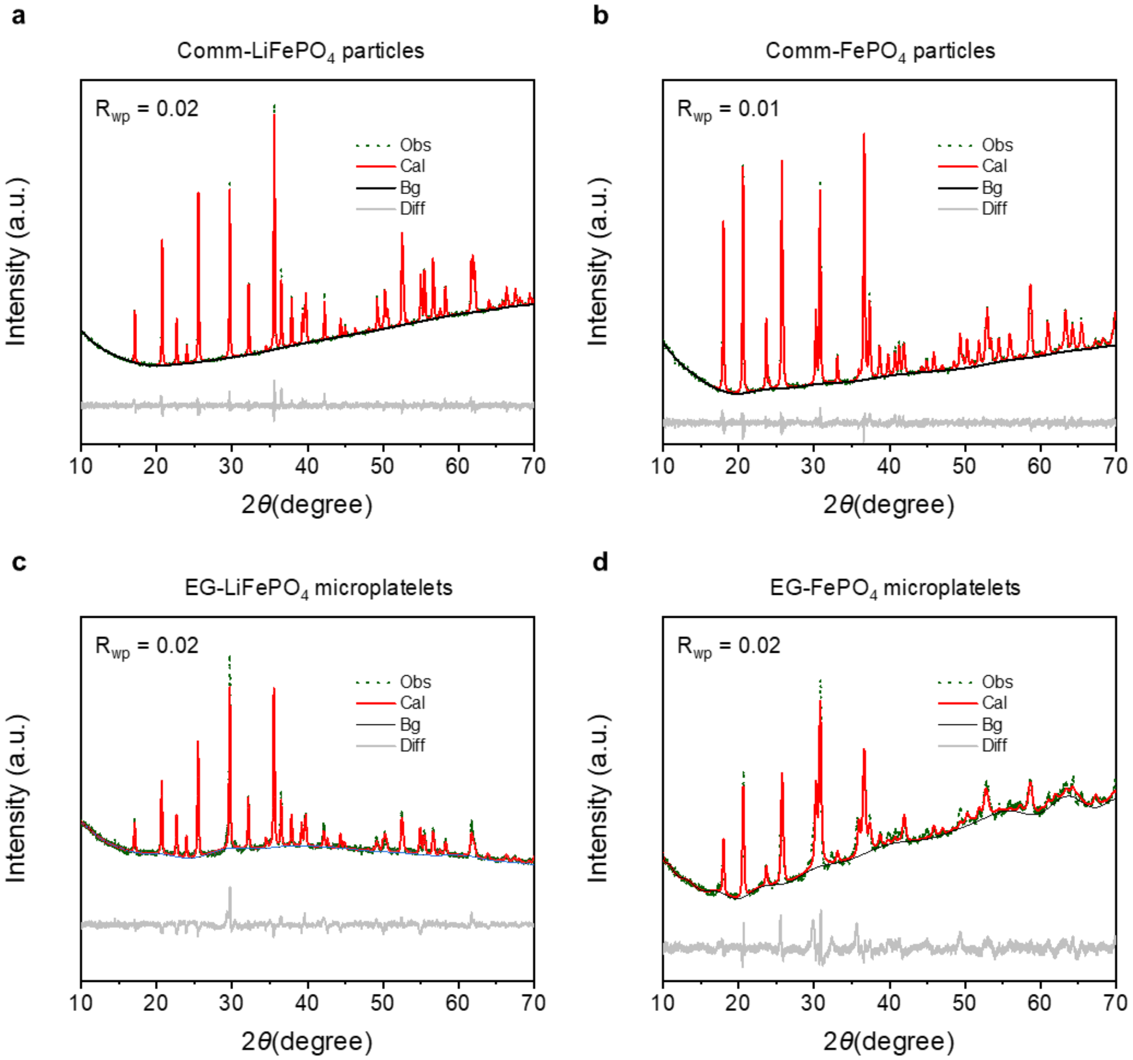

Supplementary Figure 25 Lebail refinement of (a) Comm-$LiFePO_4$ particles, (b) chemical-extracted Comm-$LiFePO_4$ particles, (c) EG-$LiFePO_4$ particles, and (d) chemical-extracted EG-$FePO_4$ particles.

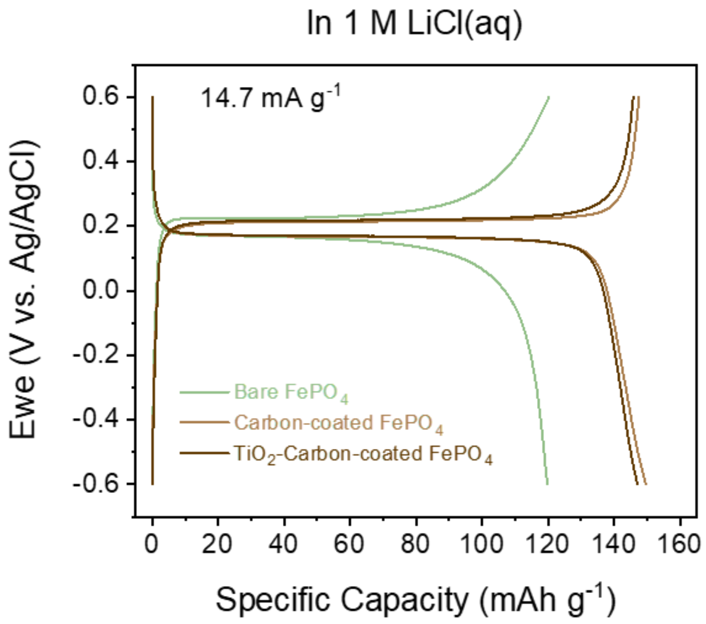

Supplementary Figure 26 Electrochemical cycling of Bare, Carbon-coated and TiO_2-Carbon-coated $FePO_4$ electrodes in 60 mL 1 M LiCl aqueous solution between -0.6 V and 0.6 V (vs. Ag/AgCl/KCl (4.0 M)) at room temperature (20 ~ 25 °C) under 0.1C (14.7 mA/g). 147 mA/g equals a rate of 1C. The counter electrode is $LiFePO_4$. N_2 (purity > 99.998%) was continuously bubbled into the solution to avoid side reactions caused from dissolved O_2.

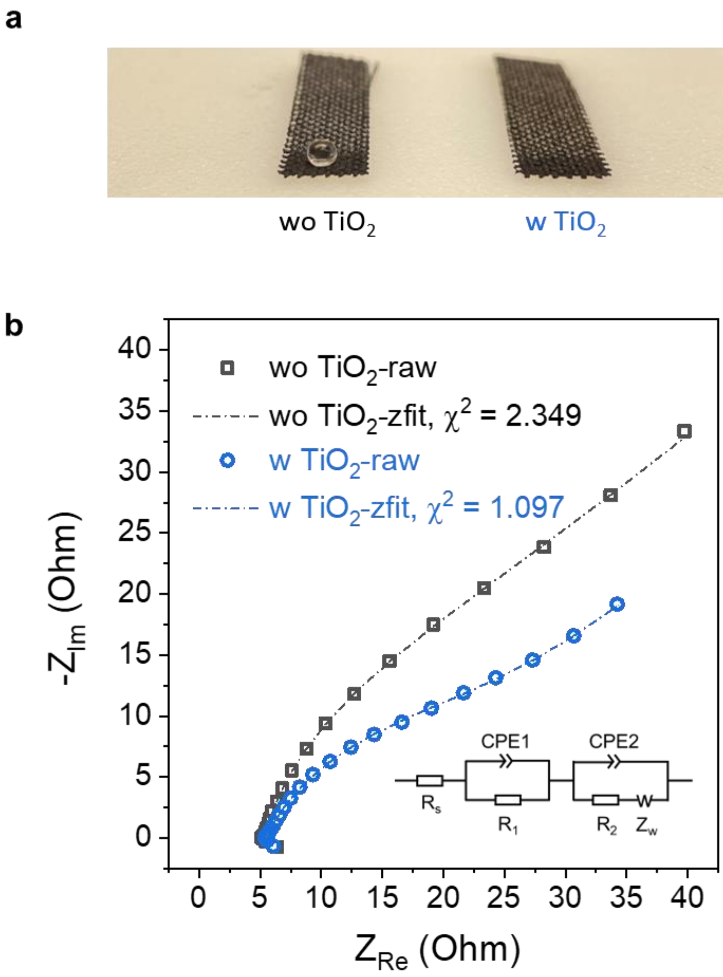

Supplementary Figure 27 (a) Photographic image showing 10 μL water dropped on the surface of the carbon-coated $FePO_4$ electrode wo/w 3nm TiO_2 coating. (b) Nyquist plots for the electrode wo/w 3nm TiO_2 obtained by electrochemical impedance spectroscopy (EIS) tests in 1 M LiCl aqueous solution with the frequency ranging from 200 kHz to 100 mHz at a 10 mV amplitude. The dot-dashed lines are the fitting curves by using the equivalent circuit, which is shown as the inset and consists of a resistor (R_s), a resistor (R_1) paralleled with a constant phase element (CPE), and a CPE parallel with a resistor (R_2) which is connected with a Warburg element (Z_w) in series.

(Noting that χ^2 gives an estimation of the distance between the real data and the simulated data. Its expression is: $\chi^2 = \sum_{i=1}^{n} \frac{|Z_{measured}(i) - Z_{simulated}(f_i,\ param)|^2}{\sigma_i^2}$

with $Z_{measured}(i)$ is the measured impedance at the f_i frequency;

$Z_{simulated}(f_i,\ param)$ is a function of the chosen model;

f_i is the frequency *i*;

$param$ is the model parameters (ex: R1, R2, C1, Q1, …);

σ_i is the standard deviation)

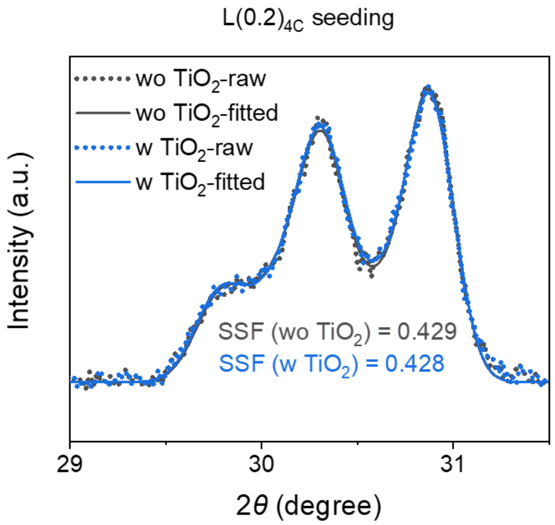

Supplementary Figure 28 XRD of the carbon-coated $FePO_4$ electrode wo/w 3nm TiO_2 coating after 20% Li seeding under 4C (588 mA/g) (Dotted lines: raw data; Solid lines: fit).

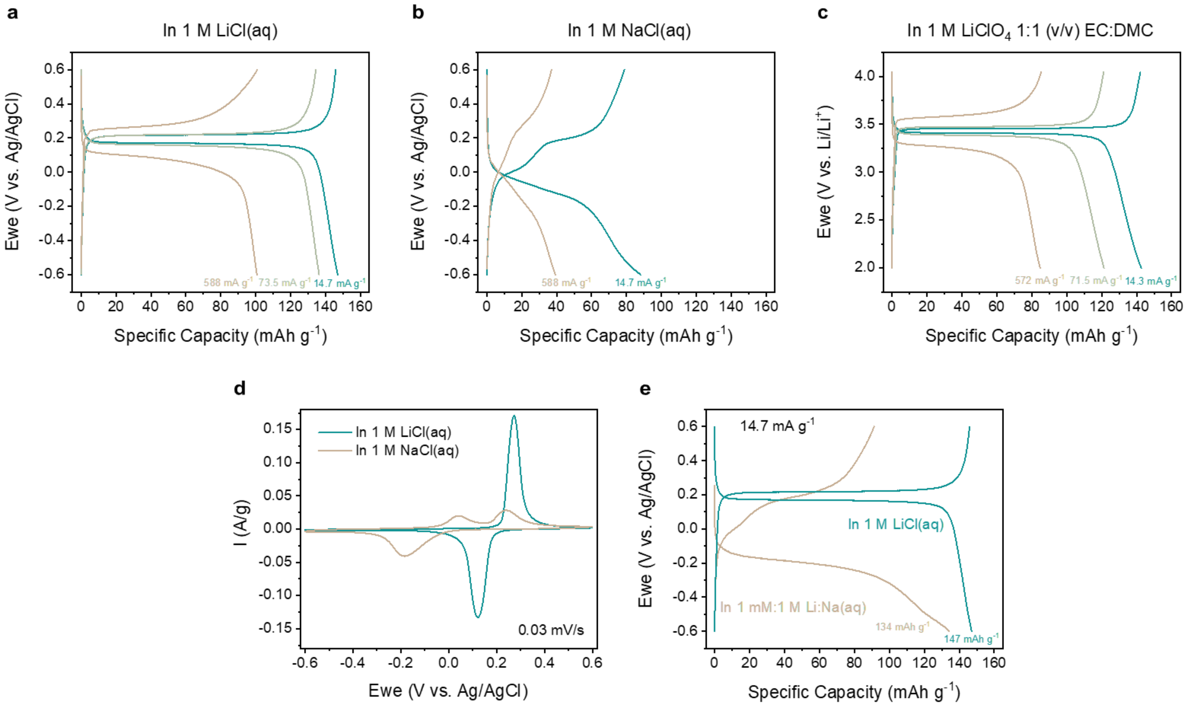

Supplementary Figure 29 Electrochemical energy storage performance of Synthesized-$FePO_4$ in aqueous electrolyte solution. Electrochemical cycling of the Synthesized-$FePO_4$ electrodes in **(a)** 60 mL 1 M LiCl aqueous solution (paired with Ag/AgCl/KCl (4.0 M) reference and $LiFePO_4$ counter electrodes) and **(b)** 60 mL 1 M NaCl aqueous solution (paired with Ag/AgCl/KCl (4.0 M) reference and $NaFePO_4$ counter electrodes) under different specific currents/C rates. 14.7 mA/g equals a rate of 0.1C, while 147 mA/g equals 1C. (**c**) Electrochemical cycling of the Synthesized-$FePO_4$ electrodes in 60 mL 1 M $LiClO_4$ 1:1 (v/v) ethylene carbonate and dimethyl carbonate electrolyte under different specific currents/C rates (paired with Ag/AgCl/(0.1M tetrabutylammonium perchlorate + 0.01M $AgNO_3$ in acetonitrile) reference and $LiFePO_4$ counter electrodes). 143 mA/g equals a rate of 1C in the organic setup. **(d)** Cyclic voltammetry (CV) tests for the Synthesized-$FePO_4$ electrodes in 60 mL 1 M LiCl/NaCl aqueous solution at a 0.03 mV/s scan rate (paired with Ag/AgCl/KCl (4.0 M) reference and $LiFePO_4$/$NaFePO_4$ counter electrodes). **(e)** Comparison of electrochemical cycling of the Synthesized-$FePO_4$ electrodes in 60 mL 1 M LiCl and 500 mL 1 mM LiCl: 1 M NaCl aqueous solutions under 0.1C (14.7 mA/g), using Ag/AgCl/KCl (4.0 M) and $LiFePO_4$/$NaFePO_4$ as the reference and counter electrodes.

(Noting that all the electrochemical operations were performed at room temperature (20 ~ 25 °C) with N_2 (purity > 99.998%) continuously bubbled into the solution from the inlet. Specifically, no climatic/environmental chamber is used.)

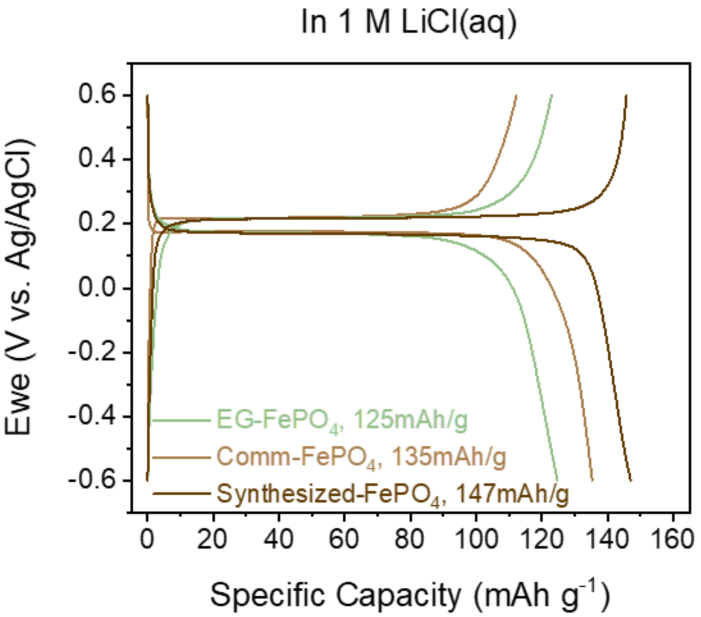

Supplementary Figure 30 Electrochemical cycling of Comm-$FePO_4$ (13.5 mA/g), Synthesized-$FePO_4$ (14.7 mA/g), and EG-$FePO_4$ (12.5 mA/g) electrodes in 60 mL 1 M LiCl aqueous solutions between -0.6 V and 0.6 V (vs. Ag/AgCl/KCl (4.0 M)) at room temperature (20 ~ 25 °C). The counter electrode is $LiFePO_4$. N_2 (purity > 99.998%) was continuously bubbled into the solution to avoid side reactions caused from dissolved O_2.

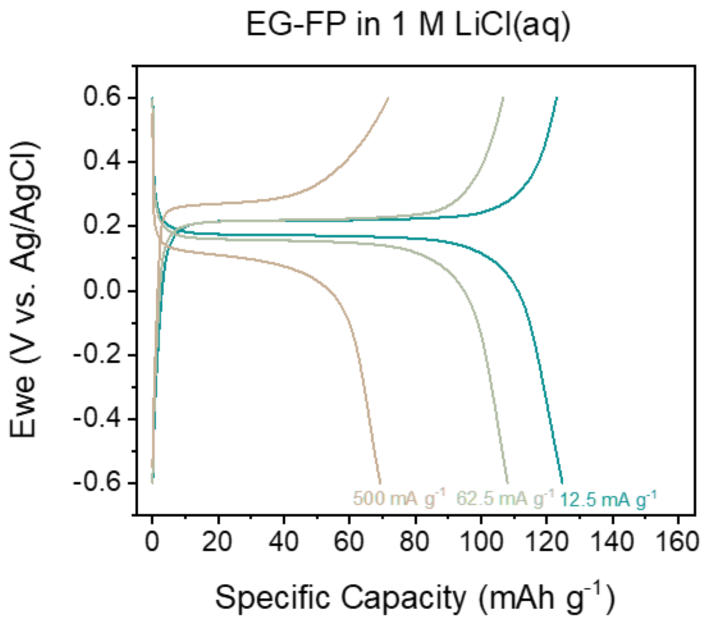

Supplementary Figure 31 Electrochemical cycling of EG-$FePO_4$ particles in 60 mL 1 M LiCl aqueous solution and under different specific currents/C rates at room temperature (20 ~ 25 °C) under 0.1C (12.5 mA/g). 125 mA/g equals a rate of 1C for EG-$FePO_4$. The counter electrode is $LiFePO_4$. The reference electrode is Ag|AgCl|KCl (4.0 M). N_2 (purity > 99.998%) was continuously bubbled into the solution to avoid side reactions caused from dissolved O_2.

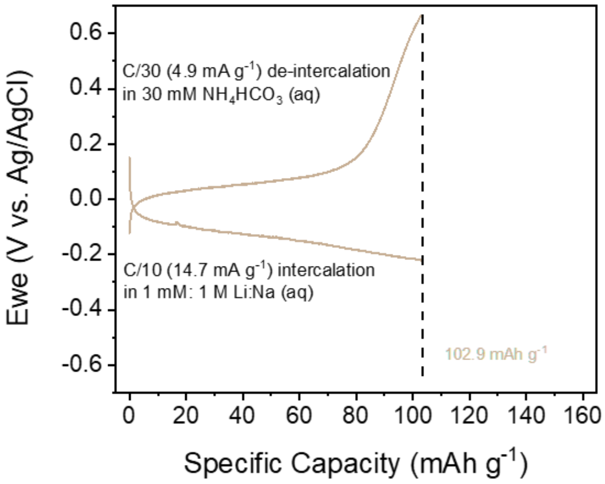

Supplementary Figure 32 One example electrochemical extraction cycle. 0.1C (14.7 mA/g) intercalation curve in 500 mL 1 mM LiCl: 1 M NaCl aqueous solution and C/30 (4.9 mA/g) de-intercalation curve in 60 mL 30 mM NH_4HCO_3 recovery solution, with the use of 70% of the total capacity (102.9 mAh/g). For both intercalation and de-intercalation, N_2 (purity > 99.998%) was continuously bubbled into the solution to avoid side reactions caused from dissolved O_2, and the reference electrode is Ag|AgCl|KCl (4.0 M) with the testing temperature at room temperature (20 ~ 25 °C). $NaFePO_4$ was used as the counter electrode during the intercalation process; while graphite rod was used as the counter electrode during the de-intercalation process.

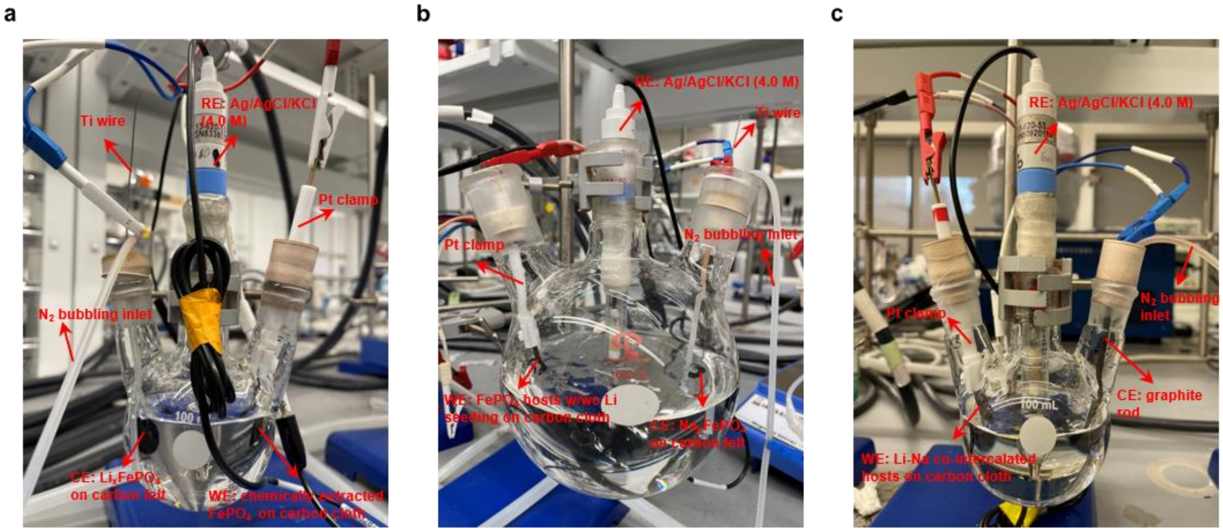

1. Li seeding in 60 mL
1 M LiCl aqueous solution

2. Li-Na co-intercalation in 500 mL
1 mM LiCl: 1 M NaCl aqueous solution

3. Li recovery in 60 mL
30 mM NH_4HCO_3 aqueous solution

Supplementary Figure 33 Detailed images of the cells used during (a) Li seeding, (b) Li-Na co-intercalation, and (c) Li recovery processes. (CE: counter electrode; RE: reference electrode; WE: working electrode)

Supplementary Table 1 Lattice parameters for potentially related Li or Na phases and the normalized values by the value of olivine $FePO_4$ (00-065-0258) phase. Lattice parameters of olivine $FePO_4$ are set as one.

	b [010] (Å)	a [100] (Å)	c [001] (Å)	α	β	γ	Volume (Å^3)	c/a	a/b	c/b
Olivine LFP (01-077-8344)	6.009 (+ 3.7%)	10.329 (+ 5.2%)	4.695 (- 1.9%)	90.00°	90.00°	90.00°	291.38 (+ 7%)	0.781	0.582	0.455
Olivine $N_{2/3}$FP (01-079-6974)	6.082 (+ 5.0%)	10.289 (+ 4.8%)	4.937 (+ 3.1%)	90.00°	90.00°	90.00°	308.96 (+ 13%)	0.812	0.591	0.480
Olivine NFP (01-079-6973)	6.219 (+ 7.3%)	10.406 (+ 6.0%)	4.947 (+ 3.3%)	90.00°	90.00°	90.00°	320.13 (+ 18%)	0.795	0.598	0.475
Olivine FP (00-065-0258)	5.795 (1.0)	9.821 (1.0)	4.787 (1.0)	90.00°	90.00°	90.00°	272.46 (1.0)	0.826	0.590	0.487
Maricite NFP (01-071-5040)	6.874 (+ 18.8%)	9.001 (- 8.4%)	5.052 (+ 5.5%)	90.00°	90.00°	90.00°	312.58 (+ 15%)	0.735	0.764	0.561

Supplementary Table 2 Lattice parameters for all the phases.

	LFP	L0.875FP	L0.750FP	L0.625FP	L0.500FP	L0.375FP	L0.250FP	L0.125FP	FP
a	10.347	10.281	10.215	10.149	10.083	10.017	9.951	9.885	9.819
b	6.007	5.981	5.955	5.929	5.903	5.877	5.850	5.824	5.798
c	4.700	4.711	4.721	4.732	4.743	4.753	4.764	4.775	4.785
α									
β	90								
γ									

Supplementary Table 3 (211) and (020) peak positions for all the phases.

Peak	LFP	L0.875FP	L0.750FP	L0.625FP	L0.500FP	L0.375FP	L0.250FP	L0.125FP	FP
(211)	29.69	29.76	29.83	29.91	29.98	30.06	30.14	30.23	30.31
(020)	29.76	29.89	30.02	30.16	30.30	30.43	30.57	30.71	30.86

Supplementary Table 4 (211) and (020) area ratios for all the phases.

Area ratio	LFP	L0.875FP	L0.75FP	L0.625FP	L0.5FP	L0.375FP	L0.25FP	L0.125FP	FP
$\frac{(211)}{(020)}$	0.23	0.249	0.268	0.286	0.305	0.324	0.343	0.361	0.38

Supplementary Table 5 Fitted phase fractions of Supplementary Figure 5 with the calculated weighted sum of Li.

	LFP	L0.875FP	L0.75FP	L0.625FP	L0.5FP	L0.375FP	L0.25FP	L0.125FP	FP	Intercalated Li	Weighted sum of Li
L(0)	0.04559	0	0.00688	0.07342	0.0824	0	0.11461	0.06579	0.61132	0	0.17471
$L(0.1)_{4C}$	0.07562	0	0.01964	0.04159	0.16232	0	0.14505	0	0.55578	0.1	0.23377
$L(0.2)_{4C}$	0.11218	0.00506	0.03933	0.06099	0.17481	0	0.11613	0.03272	0.45878	0.2	0.30475
$L(0.3)_{4C}$	0.14591	0.02684	0.01063	0.08277	0.18803	0	0.13107	0	0.41474	0.3	0.35589
$L(0.4)_{4C}$	0.16798	0.04583	0	0.12913	0.19123	0	0.10210	0.02974	0.33398	0.4	0.41365
$L(0.5)_{4C}$	0.19104	0.04145	0.07255	0.25751	0.02989	0.05795	0.04552	0.07888	0.2252	0.5	0.50058

Supplementary Table 6 Fitted phase fractions of Supplementary Figure 7 with the calculated weighted sum of Li.

	LFP	L0.875FP	L0.75FP	L0.625FP	L0.5FP	L0.375FP	L0.25FP	L0.125FP	FP	Low-Li SS	High-Li SS	Intercalated Li	Weighted sum of Li
L(0)	0	0.02175	0.02672	0	0	0	0.1191	0	0.83166	0.1191	0.04847	0	0.06962
$L(0.1)_{4C}$	0.01511	0.00562	0.08468	0	0	0	0.13104	0	0.76355	0.13104	0.0903	0.1	0.1163
$L(0.2)_{4C}$	0.06107	0.01466	0.08725	0.05607	0	0	0.14645	0	0.63449	0.14645	0.15798	0.2	0.21099
$L(0.3)_{4C}$	0.06909	0.05342	0.05712	0.12553	0	0.04728	0.11082	0.04485	0.49188	0.20295	0.23607	0.3	0.28817
$L(0.4)_{4C}$	0.08447	0.11453	0.02793	0.14864	0.04111	0.04378	0.12912	0.01258	0.39784	0.18548	0.33221	0.4	0.36936

Supplementary Table 7 Li selectivity over Na with $FePO_4$ in the literature and this work. (*: 46.4 mg Li/g $FePO_4$ is equivalent to 170 mAh/g capacity used)

Reference	Specific current during extraction (mA/g)	Capacity used during extraction (mAh/g)	Initial Li:Na (at%)	Recovered Li:Na (at%)	Selectivity
$LiFePO_4$[1]	22.2	44.4	50 mM:5 M (1:100)	5.7:1	5.70×10^{2}
		44.4	5 mM:5 M (1:1000)	1:1.8	5.60×10^{2}
		44.4	0.5 mM:5 M (1:10000)	1:4.0	2.50×10^{2}
$LiFePO_4$[2]	62.5	62.5	0.1 M:0.1 M (1:1)	225.0:1	2.25×10^{2}
		62.5	1 M:1 M (1:1)	17250.0:1	1.73×10^{4}
	6.25	62.5	42 mM:793 mM (1:19; Atacama)	9250.0:1	1.76×10^{5}
	62.5	62.5		106.5:1	2.02×10^{3}
	625	62.5		3.1:1	59
$LiFePO_4$[3]	0.184	0.66 (~215 mins operation)	5 mM:0.5 M (1:100)	2.6:1 (dissolved solid)	2.60×10^{2}
$LiFePO_4$ w/ Polydopamine coating[3]		0.66 (~215 mins operation)		43.3:1 (dissolved solid)	4.33×10^{3}
$LiFePO_4$[4]	Chemical reaction, using potassium persulfate as the oxidant	*45.3 mg Li/g $FePO_4$	60 mM:6 M (1:100)	39.2:1 (dissolved solid)	3.92×10^{3}
		*44.8 mg Li/g $FePO_4$	60 mM:3 M (1:50)	49.1:1 (dissolved solid)	2.46×10^{3}
		*35.1 mg Li/g $FePO_4$	60 mM:0.6 M (1:10)	38.4:1 (dissolved solid)	3.84×10^{2}
		*45.7 mg Li/g $FePO_4$	60 mM:4.6 M (1:77)	61.0:1 (dissolved solid)	4.70×10^{3}
		*46.4 mg Li/g $FePO_4$	200 mM:3 M (1:15)	370.0:1 (dissolved solid)	5.55×10^{3}
$LiFePO_4$ w/ TiO_2 coating[5]	34	51	0.025 mM:0.47 M (1:18500, Seawater)	1:2.18	8.49×10^{3}
	17 (P_{10s}- equivalent specific current)	51	0.025 mM:0.47 M (1:18500, Seawater)	1:1.01	1.83×10^{4}
	17 (P_{1s}- equivalent specific current)	51	0.025 mM:0.47 M (1:18500, Seawater)	1:1.11	1.67×10^{4}
	10.625	53.125	0.025 mM:0.47 M	1.01:1	1.87×10^{4}

	(P$_{10s}$R$_{2s}$- equivalent specific current)			(1:18500, Seawater)		
	17 (P$_{10s}$- equivalent specific current)	51		0.025 mM:0.47 M (1:18500, Li-Na binary)	1:1	1.85×10^4
	17 (P$_{10s}$- equivalent specific current)	51		0.235 mM:0.47 M (1:2000, Li-Na binary)	16.5:1	3.30×10^4
	17 (P$_{10s}$- equivalent specific current)	51		1.88 mM:0.47 M (1:250, Li-Na binary)	1:0 (Na below detection limit)	n.a.
**This work**	**Specific current during extraction (mA/g)**	**Capacity used during extraction (mAh/g)**	**Total capacity used (mAh/g)**	**Initial Li:Na (at%)**	**Recovered Li:Na (at%)**	**Selectivity**
	(147 mA/g = 1 C)					
L(0)-LN(0.1)$_{0.1C}$	14.7	14.7	14.7	1 mM:1 M (1:1000)	11.0:1	1.10×10^4
L(0)-LN(0.2)$_{0.1C}$	14.7	29.4	29.4		5.4:1	5.41×10^3
L(0)-LN(0.3)$_{0.1C}$	14.7	44.1	44.1		3.7:1	3.66×10^3
L(0)-LN(0.4)$_{0.1C}$	14.7	58.8	58.8		2.0:1	2.05×10^3
L(0)-LN(0.7)$_{0.01C}$	1.47	102.9	102.9		13.6:1	1.36×10^4
L(0)-LN(0.7)$_{0.1C}$	14.7	102.9			1.7:1	1.68×10^3
L(0)-LN(0.7)$_{0.2C}$	29.4	102.9			1.1:1	1.12×10^3
L(0)-LN(0.7)$_{0.5C}$	73.5	102.9			0.4:1	4.15×10^2
L(0.1)$_{4C}$-LN(0.7)$_{0.1C}$	14.7	88.2			1.6:1	1.58×10^3
L(0.2)$_{4C}$-LN(0.7)$_{0.1C}$	14.7	73.5			2.6:1	2.61×10^3
L(0.3)$_{4C}$-LN(0.7)$_{0.1C}$	14.7	58.8			3.5:1	3.50×10^3
L(0.4)$_{4C}$-LN(0.7)$_{0.1C}$	14.7	44.1			6.0:1	6.00×10^3
L(0.2)$_{4C}$-LN(0.7)$_{0.2C}$	29.4	73.5			1.6:1	1.62×10^3
L(0.2)$_{4C}$-LN(0.7)$_{0.5C}$	73.5	73.5			0.6:1	6.50×10^2
L(0.2)$_{0.1C}$-LN(0.7)$_{0.1C}$	14.7	73.5			1.9:1	1.94×10^3
L(0.2)$_{2C}$-LN(0.7)$_{0.1C}$	14.7	73.5			2.6:1	2.64×10^3
L(0.2)$_{6C}$-LN(0.7)$_{0.1C}$	14.7	73.5			2.7:1	2.72×10^3
L(0.2)$_{8C}$-LN(0.7)$_{0.1C}$	14.7	73.5			3.3:1	3.26×10^3
L(0.4)$_{0.1C}$-LN(0.7)$_{0.1C}$	14.7	44.1			2.7:1	2.74×10^3
L(0.4)$_{2C}$-LN(0.7)$_{0.1C}$	14.7	44.1			4.4:1	4.38×10^3
L(0.4)$_{6C}$-LN(0.7)$_{0.1C}$	14.7	44.1			9.0:1	9.05×10^3
L(0.4)$_{8C}$-LN(0.7)$_{0.1C}$	14.7	44.1			14.8:1	1.48×10^4

Supplementary Table 8 Summarized channel filling information. Li atoms tend to occupy more channels, with equal fractions of vacancy and Li in each channel.

Phase	Channel condition
Li0.125FP	½ quarter + ½ empty
Li0.250FP	¾ empty + ¼ full
Li0.375FP	½ half (dimer) + ½ quarter
Li0.500FP	All half filled
Li0.625FP	½ half (dimer) + ½ three quarters
Li0.750FP	All three-quarters filled
Li0.875FP	½ full + ½ three-quarters

Supplementary Table 9 Summary of calculated potential difference for Li-Na intercalation of different phases.

Li_xFePO_4	Potential difference for Li-Na intercalation [eV]
0.000	-1.1676
0.125	-1.1769
0.250	-1.2072
0.375	-1.3164
0.500	-1.3732
0.625	-1.3849
0.750	-1.3435
0.875	-1.4148

Supplementary Table 10 Li and Fe content of the chemically Li-extracted EG-$FePO_4$ hosts measured by ICP-MS. (Noting that 1. The measurement errors here denote the standard deviations of Li and Fe concentrations for each sample; 2. The average concentrations of Li and Fe are used to calculate the molar ratio for each sample; 3. Li/Fe_{ave} (at. %) denote the average Li/Fe molar ratio of the three samples.)

EG-$FePO_4$	Label	Li (ppb)	Fe (ppb)	Li/Fe (at. %)	Li/Fe_{ave} (at. %)
Raw	S1	7.405 ± 0.055	246.620 ± 1.988	24.0%	23.5%
	S2	7.843 ± 0.064	267.724 ± 1.607	23.4%	
	S3	19.095 ± 0.275	662.835 ± 4.525	23.0%	

Supplementary Table 11 Fitted phase fractions of Supplementary Figure 24 with the calculated weighted sum of Li.

EG-$FePO_4$	LFP	L0.875FP	L0.75FP	L0.625FP	L0.5FP	L0.375FP	L0.25FP	L0.125FP	FP	Intercalated Li	Weighted sum of Li
Raw	0.08255	0.10463	0.01853	0	0	0.11015	0.10872	0.245	0.33041	0	0.28711
After recovery	0.08856	0.1	0.01685	0	0	0.10857	0.12296	0.23901	0.32405	0	0.29003
$L(0.2)_{4C}$	0.1117	0.09476	0.00316	0.05839	0.03014	0.13481	0.09826	0.2001	0.26867	0.2	0.34868

Supplementary Table 12 Summarized refined lattice parameters for the three different $FePO_4$ samples.

	Comm-$LiFePO_4$	Synthesized-$LiFePO_4$	EG-$LiFePO_4$		Comm-$FePO_4$	Synthesized-$FePO_4$	EG-$FePO_4$
a (Å)	10.321	10.347	10.320	a (Å)	9.818	9.819	9.830
b (Å)	6.005	6.007	6.000	b (Å)	5.792	5.798	5.795
c (Å)	4.691	4.700	4.701	c (Å)	4.781	4.785	4.780
α	90			α	90		
β				β			
γ				γ			

Supplementary Table 13 Electrode resistances for the carbon-coated $FePO_4$ electrode wo/w 3nm TiO_2 coating obtained from equivalent circuit fitting of EIS results.

(Noting that $CPE1 = \frac{1}{Q1(j\omega)^{a1}}$, $CPE2 = \frac{1}{Q2(j\omega)^{a2}}$, $Zw = \sigma\omega^{-1/2} - j\sigma\omega^{-1/2}$ and the error is calculated using the Levenberg-Marquardt algorithm, which can be assimilated to a standard deviation. It gives an estimate of the relevancy of the parameter. If the error is very high it means that a great variation of the parameter will not affect very much the quality of the fit. Hence, the considered parameter is not critical in the minimization process.)

		wo TiO_2	w TiO_2
R_s (Ohm)		5.22 ± 0.17	5.48 ± 0.197
R_1 (Ohm)		21.06 ± 0.359	9.70 ± 6.085
R_2 (Ohm)		19.30 ± 1.388	22.64 ± 0.323
CPE1	Q1 ($F\cdot s^{(a1-1)}$)	0.02 ± 0.003	0.50 ± 0.307
	a1	0.78 ± 0.592	0.87 ± 0.592
CPE2	Q2 ($F\cdot s^{(a2-1)}$)	0.03 ± 0.001	0.01 ± 0.001
	a2	0.97 ± 0.83	0.73 ± 0.528
Zw	σ ($Ohm\cdot s^{(-1/2)}$)	24.20 ± 1.268	12.4 ± 1.502

Supplementary Table 14 Recovered $Li/(Li+Na)_{net}$ ratios of the carbon-coated $FePO_4$ electrode wo/w 3nm TiO_2 coating using 20% Li seeding (Error bars representing the standard deviation of three replicate measurements).

$L(0.2)_{4C}$-$LN(0.7)_{0.1C}$

	wo TiO_2	w TiO_2
$Li/(Li+Na)_{net}$	0.73 ± 0.01	0.74 ± 0.01

Supplementary Note 1: Calculations for $FePO_4$-$LiFePO_4$-$NaFePO_4$ ternary phase diagram

Total Energy Calculation

Total energies of structures were determined using DFT calculations with the project augmented-wave (PAW)[6] approach as implemented in the Vienna ab initio Simulation package (VASP)[7,8]. A plane wave energy cutoff of 520 eV and a Gamma-centered k-point grid with a k-point density of at least 1000/(number of atoms in unit cell) was used. The Perdew-Burke-Ernzerhof (PBE)[9] generalized-gradient approximation (GGA)[10] exchange-correlation functional with the GGA+U extension. A U value of 5.3 eV was used for Fe which was determined by the Materials Project by fitting experimental binary formation enthalpies of TM oxides[11-13]. All structures were fully optimized until the energy was converged to within 10^{-5} eV per supercell and the forces on each atom were less than 0.02 eV/Angstroms.

Structure Search

DFT energies of the $Li_xNa_yFePO_4$ ($0 \leq x+y \leq 1$) system were fit using a cluster expansion (CE) model to search for low-energy configurations given a maximum supercell size. The CE formalism is a well-established approach for studying ordering in alloys[14-17]. In the CE model the mixing enthalpies of the structures are parametrized using clusters, α. The mixing enthalpy of each structure's configuration σ is fit using a sum of weighted cluster correlation functions based on the products of occupation variables σ_i. J_α is the effective cluster interaction (ECI) for the cluster α. Using a chosen set of clusters, the energy of a structure with a configuration σ given by occupation variables σ_i is predicted using Eq. 1.

$$\Delta E(\sigma) = \sum_{\alpha} m_\alpha J_\alpha \prod_{i \epsilon \alpha'} \sigma_i \qquad (1)$$

where m_α is the multiplicity of cluster α, which is determined by the symmetry of the parent lattice. In this study two cluster expansions were fit, one for the ternary system and a second one focusing on the Li-vacancy edge of the $Li_xNa_yFePO_4$ system. In total, 506 DFT energies were calculated, with 161 of those on the Li-vacancy edge. The 506 structures show that the only intermediate structure stable with respect to the terminal compositions $LiFePO_4$, $NaFePO_4$ and $FePO_4$ is $Na_{0.66}FePO_4$. From the set of 161 structures on the Li-vacancy edge, low energy configurations with greater separation of structural Li atoms and vacancies were selected for seven intermediate Li concentrations. The selected structures were later used to calculate the difference between Li and Na intercalation potentials. The search for low energy configurations considered all supercells containing at most 86 atoms. In this work, the ICET package was used for the construction of the CE model[18]. A large cluster space (2280) with clusters up to the fourth order (quadruplets) were considered, and the Automatic relevance determination regression (ARDR) algorithm with regularization parameter, $\lambda = 15000$, was used to optimize a sparse set of clusters for the CE model.

Supplementary Note 2: Deconvolution of solid-solution fraction from diffraction patterns

To quantify the solid-solution fraction in Li-seeded $FePO_4$, we fit the obtained X-ray diffraction patterns to a number of Gaussians, following previous work[19,20]. The standard LeBail refinement was only possible for the end phases of $LiFePO_4$ and $FePO_4$ because the lattice parameters of all intermediate phases are completely interchangeable[21]. Fortunately, Vegard's law has been experimentally demonstrated to be valid in the case of Li_xFePO_4[20,22-24]. Generally, we can first calculate the corresponding lattice parameters for all intermediate phases with a linear combination of the refined end phases for $LiFePO_4$ and $FePO_4$, and then get the peak positions for all intermediate phases. Detailed steps are described below:

S1: LeBail refinement of the end phases of $LiFePO_4$ and $FePO_4$ (Supplementary Figure 4): The fitted lattice parameters for $LiFePO_4$ and $FePO_4$ are summarized in Supplementary Table 2, denoted by green and purple, respectively.

S2: Applying the Vegard's law to calculate the lattice parameters for intermediate phases (Supplementary Table 2): The deconvolution of a pattern using an infinite number of phases was assumed to be impossible. Thus, we deconvoluted the patterns for the seven intermediate phases of composition Li_xFePO_4, x = 0.125/0.250/0.375/0.500/0.625/0.750/0.875 with the space group parameter being a linear combination of the refined end phases for $LiFePO_4$ and $FePO_4$ (lattice parameters a, b, and c for space group *Pnma* for $LiFePO_4$ are 10.347, 6.007 and 4.700 Å, and those for $FePO_4$ are 9.819, 5.798 and 4.785 Å).

S3: Calculating the peak positions according to the lattice parameters in b with GSAS II software: We are using (211) and (020) peaks as characteristic peaks for the fitting, and the positions are summarized in Supplementary Table 3.

S4: Calculating (211) and (020) area ratios: The LeBail refinement of the $FePO_4$ pattern showed that the ratio between the (211) and the (020) reflection areas is 0.38 (Supplementary Figure 4). Since the (211) peak and (020) peak in $LiFePO_4$ are too close to distinguish, we use 0.23 as the area ratio according to the reference[20]. Area ratios of all intermediate phases also follow the linear combination of these two end-up phases, which are summarized in Supplementary Table 4.

S5: Normalization of areas: The scattering factors of $LiFePO_4$ and $FePO_4$ differ. Therefore, all areas were normalized to the area of $LiFePO_4$ by dividing the area of $FePO_4$ by a factor of 1.24[20].

S6: Fitting the XRD spectra with nine species: two end phases, $FePO_4$ and $LiFePO_4$, and seven intermediate phases. The difference of state of charge (SOC) between two adjacent phases is set as 12.5%. And each phase will contribute two peaks, one is (020) peak and another one is (211) peak. So totally, we need to fit the band with 18 Gaussians. We can then get all the areas of peak (211) and (020) for each Li_xFePO_4 phase (x = 0/0.125/0.250/0.375/0.500/0.625/0.750/0.875/1).

S7: The phase fraction of each Li_xFePO_4 phase ($P.F._{LixFP}$) is defined to be:

$$P.F._{Li_xFP} = \frac{\left[A_{Li_xFP}(211) + A_{Li_xFP}(020)\right] \cdot x + \frac{\left[A_{Li_xFP}(211) + A_{Li_xFP}(020)\right] \cdot (1-x)}{1.24}}{\sum\left\{\left[A_{Li_xFP}(211) + A_{Li_xFP}(020)\right] \cdot x + \frac{\left[A_{Li_xFP}(211) + A_{Li_xFP}(020)\right] \cdot (1-x)}{1.24}\right\}}$$

where $A_{Li_xFP}(211)$ is the area of Li_xFePO_4 (211) peak and $A_{Li_xFP}(020)$ is the area of the Li_xFePO_4 (020) peak.

Supplementary Note 3: Potential reasons for the deviations of calculated weighted sum of Li from fitting

As shown in Supplementary Figure 5 and Supplementary Table 5, we saw deviations in the calculated weighted sum of Li compared to our seeding range. We plotted the calculated weighted sum of Li from XRD fittings versus the electrochemically intercalated Li amount, as shown in Supplementary Figure 6. The relationship between the calculated weighted sum of Li and the depth of intercalation has good linearity ($R^2 = 0.999$). The deviation of the XRD fitted Li amount from electrochemical seeding amount indicates the possibility of unidentified system error. Even with error, this good linearity supports our analysis of the correlation of Li selectivity to Li solid solution phases since the error cannot be randomly affecting either the low-Li or high-Li solid solution phase fractions. Otherwise, there will not be a good linearity.

We think there could be two reasons that cause the deviations:

1. In order to have a quantitative measurement, we need to do LeBail refinement for the whole XRD patterns. However, the standard LeBail refinement was possible only for the end phases of LFP and FP because the lattice parameters of all intermediate phases were completely interchangeable[20]. With the help of Vegard's law, we could achieve a calculation of Li from XRD patterns. The intermediate phases do not correspond to a single phase of a specific concentration but all Li concentrations (x) in $LiFePO_4$, $0 < x < 1$ during the transition[25]. In other words, the more accurate deconvolution of the XRD intensity band requires an infinite number of phases, which is assumed to be impossible and impracticable, and could also lead to overfitting issues. Since only nine different phases of state of charge were chosen to deconvolute the XRD patterns, this simplification assigned the other intermediate phases to the nine chosen ones, which could cause deviations of the calculated weighted sum of Li.
2. We find out that the intensity contributions from the polyvinylidene difluoride (PVDF), super P, and carbon cloth substrate could also introduce deviations by raising the background intensity, especially under the low depth of intercalation. PVDF and super P are indispensable binders and conductive additives for the preparation of $FePO_4$ electrodes. The flexible carbon cloth is a good choice considering manufacturing and practical use. We then tried mirror polished glassy carbon with flatter surface as the substrate. The new glassy carbon substrate decreases the deviations caused by porous structures of carbon cloth. As shown in Supplementary Figure 7 and Supplementary Table 6, we achieved a better agreement between the calculated weighted sum of Li and the depth of intercalation, which also shows a good linear relationship in Supplementary Figure 8a. We also witnessed a similar monotonically increasing trend of high-Li SS phases with increased seeding range under 4C (588 mA/g) in Supplementary Figure 8b, while the low-Li SS phases still did not correlate with the increasing Li seeding range.

At this stage, although we witnessed some deviations of the calculated weighted sum of Li from deconvolution results, the general trend is repeatable and reasonable. Moreover, for the consistency of comparisons and practical feasibility, all the samples are tested on carbon cloth substrate unless specified.

Supplementary Note 4: Calculating Li-Na potential difference with respect to solvated ions

For the structures on the Li-vacancy edge of the $Li_xNa_yFePO_4$ system the difference in potential for intercalating Li vs Na was calculated by determining the energy contribution from the electrochemical metal-ion insertion reaction shown in the equation below:

$$A_{n-x}FePO_4(s) + xA^+(solv) + xe^- \xrightarrow{\Delta G^{cathode}} A_nFePO_4(s)$$

The preference of Li vs. Na is compared by calculating the concentration corrected chemical potential of Li and Na[26]. To be specific,

$$\Delta G^{cathode}_{solvated,A^+} = -x\Delta G^{A^+}_{solv} - xIE - \Delta G^{cathode}_{atom,A} + 0.059\log(\frac{1}{[A]^x})$$

where $\Delta G^{cathode}_{atom,A}$ is the energy to extract an isolated atom A from the cathode, IE is the ionization energy of A, and $\Delta G^{A^+}_{solv}$ is the solvation energy of A^+. Precisely, according to the literature, the ionization energy for Li and Na are 5.39171 eV and 5.13908 eV respectively[27]. Meanwhile, the solvation energy for Li^+ and Na^+ are 5.389 eV and 4.198 eV respectively[28]. The Li and Na potentials were calculated at 8 different Li-ion concentrations (0, 0.125, 0.25, 0.375, 0.5, 0.625, 0.75, and 0.875). At each composition, among the low energy configurations the configurations with greater separation of structural Li atoms and vacancies were selected. This is because the thermodynamically stable state of these compositions is a decomposition into $LiFePO_4$ and $FePO_4$. For each selected configuration a single Li or Na atom was placed on a vacancy site. If there were vacancy sites with different number of first, second, third structural Li nearest neighbors, two different calculations were performed. One where the added Li or Na atom was placed in order to maximize the proximity of structural Li atoms, and another where the Li or Na atom was placed as far away from the structural Li atoms as possible. Of the two resulting energies, the one with the lower energy was used for the Li and Na potential calculation. All configuration corresponded to 56 atom supercells, if all vacancies were filled, therefore with the addition of a single Li or Na atom x in the above equations is 0.125.

Then the Li-Na potential difference for each phase can be calculated using the following equation:

$$\Delta G^{Li_xFePO_4}_{Li-Na} = \Delta G^{Li_xFePO_4}_{solvated,Li^+} - \Delta G^{Li_xFePO_4}_{solvated,Na^+}$$

The calculated Li-Na intercalation potential differences for each phase are summarized in Supplementary Table 9. We also provided channel filling information for each intermediate phase in Supplementary Table 8 for reference. More negative Li-Na potential difference shows that the Li-ion intercalation is preferred to Na-ion intercalation.

Supplementary Note 5: Roles of surface carbon and TiO_2 coatings

Surface carbon coating was conducted for each $FePO_4$ sample by sugar pyrolysis, followed by 3 nm TiO_2 coating over the entire electrode. 3 nm TiO_2 was coated onto the $FePO_4$ electrodes using atomic layer deposition (ALD) at 100°C, 0.645 Å/cycle with tetrakis(dimethylamido)titanium (IV) and H_2O as precursors. We discussed the roles of surface carbon and TiO_2 coatings below for reader's information:

1. The battery cycling performance comparison among Bare, Carbon-coated, and TiO_2-Carbon-coated $FePO_4$ electrodes is summarized in Supplementary Figure 26. Under 0.1C (14.7 mA/g), the TiO_2-Carbon-coated $FePO_4$ electrode delivered a similar specific capacity (147 mAh/g) with only Carbon-coated $FePO_4$ electrode (151 mAh/g), while the Bare-$FePO_4$ electrode without either coating delivered a much worse specific capacity (120 mAh/g; Note: bare particles were annealed under the same condition without mixing with sucrose). Surface carbon coating does help improve the performance of $LiFePO_4$ electrodes.

2. For the role of TiO_2 coating, as shown in Supplementary Figure 27a, without TiO_2 coating, the carbon-coated $FePO_4$ together with carbon cloth substrate is highly hydrophobic. After coating the electrode with TiO_2, the 10 µL water immediately infiltrated the electrode surface. Several pre-wetting steps can be avoided using TiO_2-Carbon-coated $FePO_4$ electrodes. We also conducted electrochemical impedance spectroscopy measurements in 1M LiCl aqueous solution. Supplementary Figure 27b shows the Nyquist plots for the carbon-coated $FePO_4$ wo/w the 3 nm TiO_2 coating with the equivalent circuit shown in the inset. The resistor R_s corresponds to the electrolyte resistance. The resistors R_1 and R_2 paralleled with the constant phase element (CPE) account for the contact impedance and charge transfer impedance, respectively. The ion diffusion in the host material is described with the Warburg element (Z_w). As shown in Supplementary Figure 27b, the data from the equivalent circuit well fit the impedance data for both electrodes. The values for the different resistances obtained from the fitting are listed in Supplementary Table 13. As shown in the table, the electrolyte resistance (R_s) and charge transfer impedance (R_2) are almost the same for the carbon-coated $FePO_4$ wo/w the TiO_2 coating. The contact impedance (R_1) of the electrode without TiO_2 coating is more than double the value of the electrode with TiO_2 coating. Therefore, the TiO_2 coating can reduce the contact resistance as well.

3. We further evaluated the effect of TiO_2 coating on the seeding process. Supplementary Figure 28 shows the XRD patterns of the carbon-coated $FePO_4$ electrode wo/w TiO_2 coating after 20% Li seeding under 4C (588 mA/g). There is little difference for both obtained and fitted patterns. The calculated solid solution fraction (SSF) for the electrode without or with TiO_2 coating are 0.429 and 0.428, respectively, which is similar. And the recovered Li/(Li+Na) ratios for the following Li extraction step are almost the same (Supplementary Table 14). Without TiO_2, $Li/(Li+Na)_{net}$ is 0.73 ± 0.01, and with TiO_2, $Li/(Li+Na)_{net}$ is 0.74 ± 0.01. Therefore, the above results show that the TiO_2 coating will not affect SSF generated in the seeding process, as well as the following Li extraction performance. Long term corrosion resilience can be a benefit of TiO_2 coating.

Supplementary References

1 Pasta, M., Battistel, A. & La Mantia, F. Batteries for lithium recovery from brines. *Energy Environ. Sci.* **5**, 9487-9491, doi:10.1039/C2EE22977C (2012).

2 Trocoli, R., Battistel, A. & La Mantia, F. Selectivity of a Lithium-Recovery Process Based on $LiFePO_4$. *Chemistry-a European Journal* **20**, 9888-9891, doi:10.1002/chem.201403535 (2014).

3 Kim, J.-S. *et al.* An Electrochemical Cell for Selective Lithium Capture from Seawater. *Environmental science & technology* **49**, doi:10.1021/acs.est.5b00032 (2015).

4 Intaranont, N., Garcia-Araez, N., Hector, A. L., Milton, J. A. & Owen, J. R. Selective lithium extraction from brines by chemical reaction with battery materials. *Journal of Materials Chemistry A* **2**, 6374-6377, doi:10.1039/C4TA01101E (2014).

5 Liu, C. *et al.* Lithium Extraction from Seawater through Pulsed Electrochemical Intercalation. *Joule* **4**, 1459-1469, doi:10.1016/j.joule.2020.05.017 (2020).

6 Blöchl, P. E., Jepsen, O. & Andersen, O. K. Improved tetrahedron method for Brillouin-zone integrations. *Phys. Rev. B* **49**, 16223-16233, doi:10.1103/PhysRevB.49.16223 (1994).

7 Kresse, G. & Furthmüller, J. Efficient iterative schemes for ab initio total-energy calculations using a plane-wave basis set. *Phys. Rev. B* **54**, 11169-11186, doi:10.1103/PhysRevB.54.11169 (1996).

8 Kresse, G. & Furthmüller, J. Efficiency of ab-initio total energy calculations for metals and semiconductors using a plane-wave basis set. *Computational Materials Science* **6**, 15-50, doi:10.1016/0927-0256(96)00008-0 (1996).

9 Perdew, J. P., Burke, K. & Ernzerhof, M. Generalized Gradient Approximation Made Simple. *Physical Review Letters* **77**, 3865-3868, doi:10.1103/PhysRevLett.77.3865 (1996).

10 Perdew, J. P., Ernzerhof, M. & Burke, K. Rationale for mixing exact exchange with density functional approximations. *The Journal of Chemical Physics* **105**, 9982-9985, doi:10.1063/1.472933 (1996).

11 Anisimov, V. I., Aryasetiawan, F. & Lichtenstein, A. I. First-principles calculations of the electronic structure and spectra of strongly correlated systems: the LDA+U method. *Journal of Physics: Condensed Matter* **9**, 767-808, doi:10.1088/0953-8984/9/4/002 (1997).

12 Zhou, F., Maxisch, T. & Ceder, G. Configurational Electronic Entropy and the Phase Diagram of Mixed-Valence Oxides: The Case of Li_xFePO_4. *Physical Review Letters* **97**, 155704, doi:10.1103/PhysRevLett.97.155704 (2006).

13 Jain, A. *et al.* Commentary: The Materials Project: A materials genome approach to accelerating materials innovation. *APL Materials* **1**, 011002, doi:10.1063/1.4812323 (2013).

14 Sanchez, J. M., Ducastelle, F. & Gratias, D. Generalized cluster description of multicomponent systems. *Physica A: Statistical Mechanics and its Applications* **128**, 334-350, doi:https://doi.org/10.1016/0378-4371(84)90096-7 (1984).

15 Chen, W., Schmidt, D., Schneider, W. F. & Wolverton, C. First-principles cluster expansion study of missing-row reconstructions of fcc (110) surfaces. *Phys. Rev. B* **83**, 075415, doi:10.1103/PhysRevB.83.075415 (2011).

16 Chen, W., Schmidt, D., Schneider, W. F. & Wolverton, C. Ordering and Oxygen Adsorption in Au–Pt/Pt(111) Surface Alloys. *The Journal of Physical Chemistry C* **115**, 17915-17924,

doi:10.1021/jp205995j (2011).

17 Chen, W., Dalach, P., Schneider, W. F. & Wolverton, C. Interplay between Subsurface Ordering, Surface Segregation, and Adsorption on Pt–Ti(111) Near-Surface Alloys. *Langmuir* **28**, 4683-4693, doi:10.1021/la204843q (2012).

18 Ångqvist, M. *et al.* ICET – A Python Library for Constructing and Sampling Alloy Cluster Expansions. *Advanced Theory and Simulations* **2**, 1900015, doi:https://doi.org/10.1002/adts.201900015 (2019).

19 Li, Y. *et al.* Fluid-enhanced surface diffusion controls intraparticle phase transformations. *Nat. Mater.* **17**, 915-922, doi:10.1038/s41563-018-0168-4 (2018).

20 Hess, M., Sasaki, T., Villevieille, C. & Novák, P. Combined operando X-ray diffraction–electrochemical impedance spectroscopy detecting solid solution reactions of $LiFePO_4$ in batteries. *Nature Communications* **6**, 8169, doi:10.1038/ncomms9169 (2015).

21 Bail, A. Whole powder pattern decomposition methods and applications: A retrospection. *Powder Diffraction* **20**, 316-326 (2005).

22 Delacourt, C., Poizot, P., Tarascon, J.-M. & Masquelier, C. The existence of a temperature-driven solid solution in Li_xFePO_4 for $0 \le x \le 1$. *Nat. Mater.* **4**, 254-260, doi:10.1038/nmat1335 (2005).

23 Gibot, P. *et al.* Room-temperature single-phase Li insertion/extraction in nanoscale Li_xFePO_4. *Nat. Mater.* **7**, 741-747, doi:10.1038/nmat2245 (2008).

24 Sharma, N. *et al.* Direct Evidence of Concurrent Solid-Solution and Two-Phase Reactions and the Nonequilibrium Structural Evolution of $LiFePO_4$. *Journal of the American Chemical Society* **134**, 7867-7873, doi:10.1021/ja301187u (2012).

25 Orikasa, Y. *et al.* Direct Observation of a Metastable Crystal Phase of Li_xFePO_4 under Electrochemical Phase Transition. *Journal of the American Chemical Society* **135**, 5497-5500, doi:10.1021/ja312527x (2013).

26 Ong, S. P. *et al.* Voltage, stability and diffusion barrier differences between sodium-ion and lithium-ion intercalation materials. *Energy Environ. Sci.* **4**, 3680-3688, doi:10.1039/c1ee01782a (2011).

27 Haynes, W. M. *CRC Handbook of Chemistry and Physics*. 91st edn, (CRC Press, 2010).

28 Marcus, Y. *Ion Solvation*. (John Wiley & Sons, 1985).
